# Assignment of PolyProline II Conformation and Analysis of Sequence – Structure Relationship

**DOI:** 10.1371/journal.pone.0018401

**Published:** 2011-03-31

**Authors:** Yohann Mansiaux, Agnel Praveen Joseph, Jean-Christophe Gelly, Alexandre G. de Brevern

**Affiliations:** 1 INSERM, UMR-S 665, Dynamique des Structures et Interactions des Macromolécules Biologiques (DSIMB), Paris, France; 2 Université Paris Diderot - Paris 7, Paris, France; 3 Institut National de la Transfusion Sanguine (INTS), Paris, France; Massachusetts Institute of Technology, United States of America

## Abstract

**Background:**

Secondary structures are elements of great importance in structural biology, biochemistry and bioinformatics. They are broadly composed of two repetitive structures namely α-helices and β-sheets, apart from turns, and the rest is associated to coil. These repetitive secondary structures have specific and conserved biophysical and geometric properties. PolyProline II (PPII) helix is yet another interesting repetitive structure which is less frequent and not usually associated with stabilizing interactions. Recent studies have shown that PPII frequency is higher than expected, and they could have an important role in protein – protein interactions.

**Methodology/Principal Findings:**

A major factor that limits the study of PPII is that its assignment cannot be carried out with the most commonly used secondary structure assignment methods (SSAMs). The purpose of this work is to propose a PPII assignment methodology that can be defined in the frame of DSSP secondary structure assignment. Considering the ambiguity in PPII assignments by different methods, a consensus assignment strategy was utilized. To define the most consensual rule of PPII assignment, three SSAMs that can assign PPII, were compared and analyzed. The assignment rule was defined to have a maximum coverage of all assignments made by these SSAMs. Not many constraints were added to the assignment and only PPII helices of at least 2 residues length are defined.

**Conclusions/Significance:**

The simple rules designed in this study for characterizing PPII conformation, lead to the assignment of 5% of all amino as PPII. Sequence – structure relationships associated with PPII, defined by the different SSAMs, underline few striking differences. A specific study of amino acid preferences in their N and C-cap regions was carried out as their solvent accessibility and contact patterns. Thus the assignment of PPII can be coupled with DSSP and thus opens a simple way for further analysis in this field.

## Introduction

The three dimensional structures of proteins are of great help to understand the precise details of its biological function. Contrary to the earlier views, the first low resolution model of myoglobin exhibited high complexity and a definite lack of symmetry [1]. In spite of the global complexity, Pauling and Corey had proposed two types of regularities in the local backbone conformation [2], [3]. The first one constitutes the α-helix conformation which was estimated to be stable and favorable on the basis of accurate geometrical parameters derived from small molecule crystal structures [4]. The second one is the β-sheet which was made of extended anti-parallel or parallel strands stabilized by backbone hydrogen bonds between them [5]. The high frequency of α-helices and β-sheets observed in experimentally determined structures [6] has led to the concept of ‘secondary structures’ which describes these local backbone regularities in the protein structure. Basically the secondary structure description is composed mainly of α-helix, β-strand and a state corresponding to other regions in the backbone, the coil. The structure descriptions are often limited to these three classes. With availability of a large number of experimentally determined protein structures, it is becoming obvious that other backbone conformations are also favored in proteins. The α-helices are not the only helical conformation and are often not linear [7], [8], the β-sheets also show irregularities [9], [10] and the coil is in fact, not strictly random.

The class of β-turns is of particular interest. In the late 60 s, Venkatachalam discovered the existence of these small local folds that are characterized by the reversal of polypeptide chain and stabilized by a hydrogen bond between the first and the last residue [11]. These β-turns are part of a more general class, known as tight turns, which are characterized by precise dihedral angle values of their central residues and a short distance between the extremities [12]. The latter has been shown to play important structural and functional roles [13].

Apart from turns, the other characterized secondary structure is the PolyProline II (PPII) helix. The PPII helices correspond to a unique local fold [14]. They were discovered more than 50 years ago in fibrous proteins [15], [16], as they contribute to coiled coil supersecondary structures formation . Later they were also found to occur in numerous globular proteins. PPII helix is a left-handed helical structure with an overall shape resembling a triangular prism [17], [18]. With a helical pitch of 9.3 Å/turn, each turn constituting of 3 residues, it forms an extended helix. This conformation is characterized by recurrent trans isomers of peptide bonds and (φ, ψ) values of −75° and +145° respectively, the dihedral angles being a characteristic of β-strands As noted by G. Rose, considering the hydrogen-bonded β-turns [11] and PPII [16] along with the classical secondary structure result in the assignment of 80% of the all amino acids to a regular backbone conformation [19].This rises to 90%, in a more recent study where the turns are defined in slightly different way [20].

Nonetheless, when compared to α-helices, β-sheets and turns, analysis of PPII has not gained wide interest, mainly due to three factors: (*i*) PPII has a low frequency of occurrence, (*ii*) PPII conformation is not stabilized by a strong hydrogen bond pattern, thus considered as an unstable conformation and, (*iii*) only a few methods for PPII assignment are available and these methods use different assignment parameters resulting in variable assignments. Also, PPII helices are not assigned by the widely used Secondary Structure Assignment Method (SSAM): DSSP [21].

Adzhubei and Sternberg in their first systematic search, found 96 PPII helices in a databank of 80 proteins [22]. They were surprisingly common. Even if they are called polyproline for historical reasons, they are not only composed of Proline successions, some PPII helices have no Proline at all [22], [23], [24], [25], [26] , *e.g.*, short stretches of poly-glutamines were found to form PPII conformation [27]. Hollingsworth, Berkholz and Karplus recently proposed that its common name could be changed to a more general form, *i.e.*, “polypeptide-II”. This would maintain the familiar acronym, avoid the misleading association with only Proline, and be consistent with the observation that it is a prominent conformation in unfolded polypeptide chains [28].

These PPII helices are highly solvent-exposed and tend to have high crystallographic temperature factors [22]. Moreover PPII are not stabilized by salt bridges [29]. It has been suggested that PPII helices could be stabilized by water mediated main chain hydrogen bonds (in the absence of main chain-main chain H-bonds), as they also tend to have a regular pattern of hydrogen bonds with water [30]. Several studies suggest that peptide-solvent interaction is a major determinant of PPII conformation [31], [32], [33]. However, the preference for polyproline II conformation is also reported to be independent of the degree of solvation [34]. Avbelj and Baldwin noticed that solvation strongly affects preferences for different backbone conformations. The dependence of backbone preference on solvation might explain why Alanine favors PPII conformation whereas Valine favors extended structure [35]. Stapley and Creamer suggested that local side-chain to main-chain hydrogen bonds are also important in stabilizing PPII helices [24]. Cubellis and co-workers recently highlighted that PPII helices are stabilized by non-local interactions [36]. PPII do not display strong sequence propensities in contrast to the other extended conformations, such as β-strands [37]. The non-local stabilization of hydrogen-bond donors and acceptors does, however, result in PPII conformations being well suited for participating in protein-protein interactions. They are also suspected to have a role in amyloid formation [38], [39] and nucleic acid binding [40]. Hence, a recent study shows its importance in Duchenne muscular dystrophy [41]. Several studies have also focused on the extremities of PPII. It has been proposed that PPII might interrupt in the formation of β-sheet which is prone to aggregation [42], due to its particular geometry with the neighboring amide bond [43]. They could have a key role in the folding process [44]; the concentration of residues in the PPII conformational space lowers the entropy of the unfolded protein chain and thus facilitates folding (under appropriate conditions) [45], [46]. Recently, the number of studies on PPII conformations has increased [19], [36], [47], [48], [49], especially in the field of molecular dynamics [19], [32], [45], [50].

Numerous approaches for secondary structure assignment that rely on different descriptors, exist (see Table 1 of [51]). DSSP [21] remains the most widely used SSAM. It identifies the secondary structures on the basis of particular hydrogen bond patterns detected from the protein geometry, with the help of an electrostatic model. DSSP is used for assigning secondary structures for the protein structures deposited in the Protein DataBank (PDB) [6], [52].

**Table 1:** Analysis of PPII properties.

f(PPII)	all (%)	in DSSP coil (%)	av. len.	in PPII^DSSP^ (%)
PROSS	10.10	6.70	1.35	65.9
XTLSSTR	6.80	4.18	2.63	56.0
SEGNO	3.97	2.58	2.58	45.6
mean	6.95	4.48		
DSSP with PPII^DSSP^	5.11	5.11	3.24	100.0

**Figure 1:**
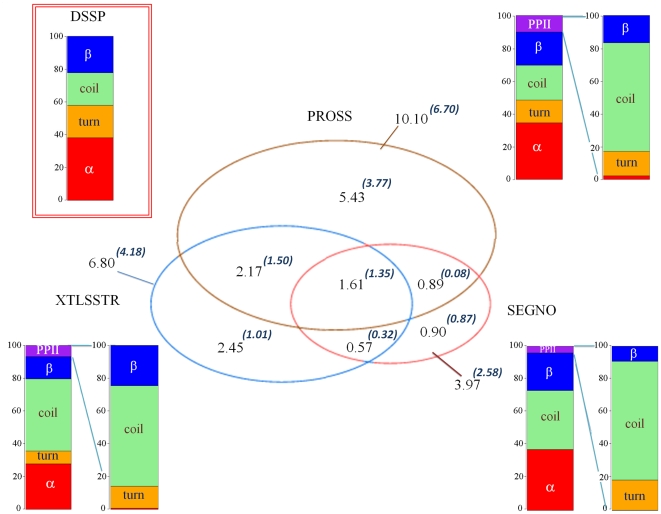
PPII distribution. The Venn diagram gives the confusion between PPII assignments by the three SSAMs, namely PROSS, XTLSSTR and SEGNO. Inside different regions of the diagram is given two percentages x.xx*^(y.yy)^*, the first percentage x.xx% correspond to the PPII frequency observed while y.yy gives the frequency in the DSSP coil alone. The secondary structure frequencies are given as a barplot in 4 states for DSSP, and 5 states for the other three assignments. The correspondence of PPII residues with the assignments by DSSP is also given.

The PPII frequency of the 3 SSAMs (see Figure 1) and the novel PPII^DSSP^ are given with their average PPII length (*av. len.*) and the percentage of PPII assigned by PPII^DSSP^ which are in common with the other SSAM assignments.

Currently, only three publicly available SSAMs assign PPII, namely XTLSSTR [53], PROSS [54] and SEGNO [55]. XTLSSTR uses all the backbone atoms to compute two angles and three distances [53], which forms the basis of assignments. Assignments made by PROSS and SEGNO are based solely on backbone angles, mainly involving the ϕ and ψ dihedral angles.

The purpose of this work is to propose simple rules to assign PPII based on a classical secondary structures assignment carried out using DSSP. We have compared the assignment of the three available PPII assignment methods and specifically analyzed the distribution of PPII based on these assignments. Then, we propose a rule to assign PPII within the coil assigned by DSSP; such that there is a good concordance with the other assignments. Sequence – structure relationships of these PPIIs are also analyzed to study amino acid preferences and to ensure a good agreement with the previous studies [56]. In the same way, a specific analysis of sequence-structure relationships on capping regions was also carried out. Residue accessibility and contacts in the PPII helices were also studied as they are considered to be accessible for different interactions. The behaviour of PPII has also been analyzed at the light of a structural alphabet [56], [57] named Protein Blocks [58], [59], [60]. This gives a more accurate picture on the local structures associated with PPII.

## Results

### Comparison of the different SSAMs

A non-redundant databank of protein structures has been extracted from the PDB [6]. The list of protein structures has been obtained from PISCES database [61], [62], which is generated based on the following criteria : resolution less than 2.5 Å, R factor below 0.2 and no proteins share more than 30% sequence identity. As assessed earlier [51], SSAMs have only about 80% of consensus between them. We have re-computed an agreement rate *C*_3_ between the four SSAM assignments used in this study (see Figure S1). The results were in agreement with previous studies [51], [55], [63], [64]: the secondary structure assignment by XTLSSTR had the lowest concordance with others, having *C*_3_ values of 77.4, 73.5 and 73.8% with DSSP, PROSS and SEGNO respectively (mainly due to the highest frequency of assigned α-helices, as already observed in [65]). The assignment made by SEGNO remains closest to DSSP with a *C*_3_ of 88.9%. For PROSS, which was not used in the earlier analysis, the *C*_3_ values are around 82% (corresponding to classical values [51], [66], [67]). These results show that the two closest SSAMs are DSSP and SEGNO while PROSS is a little far away and XTLSSTR is even more distant.

### PPII

Figure 1 summarizes most of the information on PPII distribution (see also Table 1). The frequencies of classical secondary structures (see Figure S2 and S3) and especially of PPII are rather different for each SSAM. Frequencies of secondary structures assigned by DSSP and SEGNO are quite similar (except that SEGNO does not assign turns). Assignments made by PROSS have relatively similar frequencies when compared to that of DSSP, with a slightly lower frequency of turns [65]. XTLSSTR assigns more coil and less repetitive structures when compared to DSSP.

The percentage of PPII is quite significant, starting from 3.97% for SEGNO, 6.80% for XTLSSTR and 10.10% for PROSS. A ratio of 2.5 is thus observed between PPII assignments. However 1/3^rd^ of the PPII assigned by each SSAM is not associated to DSSP coil, but is shared between turns and β-strands. Considering only the DSSP coil regions, the PPII frequencies range only from 2.58% for SEGNO to 6.70% for PROSS. As seen in the Venn diagram, the consensus of PPII assignment is more limited, *i.e.*, only 1.35% of the residues amino acids are assigned as PPII by all the 3 SSAMs. It represents 52% of PPII assigned by SEGNO, 32% by XTLSSTR and 20% by PROSS. If only two SSAMs are taken into account, the consensus in the region of DSSP coil goes up to 3.25%. Thus the average frequency of PPII is about 6.95%, and it is about 4.48% in the DSSP coil. Hence, no simple consensus emerges from the analysis of the different PPII assignments (see [51], [63], [65], [67]).

### Definition of the PPII assignment based on DSSP

#### Choice of dihedrals

Seeking hints from literature [22] [37] and the above analysis of previous methods [53], [54], [55], we propose a simple rule for assigning PPII conformation for the residues in the coil assigned by DSSP. For this purpose, we have used the dihedral angles (.φ+/−.ε, ψ+/−ε) to delineate the PPII space. The canonical (.φ, ψ) values of −75° and +145° have been selected as the core of PPII region, and an ε increasing by steps of 1°. Mean φ value of PPII assigned by the SSAMs equals 75.6° while mean ψ value is slightly different from the canonical value, *i.e.*, 136.9°.

**Figure 2:**
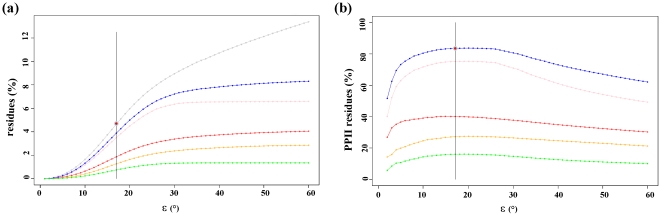
Deriving PPII assignment criterion: Choice of epsilon. (a) shows the percentage of residues found for ε ranging from 0 to 60°. The percentage of amino acids associated to DSSP coil is shown in grey, PII assigned by PROSS is indicated in pink, assignment by XTLSSTR is in red and that of SEGNO in orange. In blue is shown the percentage of residues considered as PPII at least by one of the three SSAMs and the green plot indicates percentage of common assignments. (b) The same information is given as the percentage of PPII residues (in regards to DSSP coil). In the initial search, the epsilon chosen corresponds to 17°, it is indicated by a black line.

The value of ε is chosen such that equilibrium is reached between the number of amino acids assigned as PPII by one of the SSAMs and the residues not assigned as PPII in the DSSP coil. The higher the ε, the higher is the number of PPII assigned by one of the three SSAMs and higher is the number of amino acids not assigned as PPII by one or another. Figure 2a shows the distribution of PPII assigned in DSSP coil and Figure 2b gives the corresponding percentage in DSSP coil. With an ε of 17°, we have the highest percentage of amino acids assigned as PPII by SEGNO, XTLSSTR and PROSS, within the DSSP coil. Moreover, the corresponding percentage assigned (*i.e.*, 4.9%) is close to the average occurrence of PPII (see previous sections). However, some PPII helices are only one residue long. It is mainly due to the delimitation of DSSP coil (see Table 1).

#### Additional constraint

**Figure 3:**
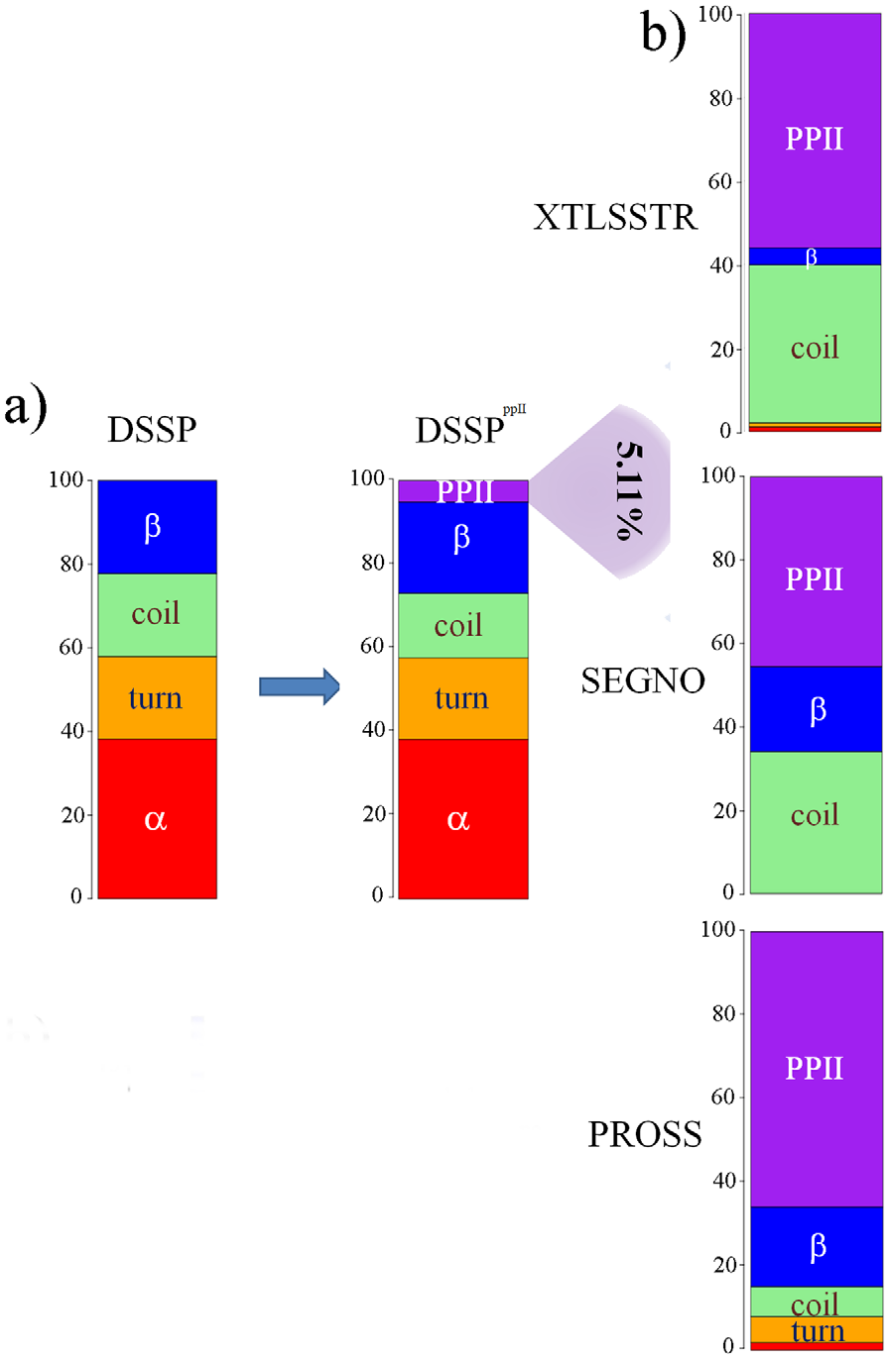
PPIIs distribution with the length constraint. PPII residues assigned in DSSP coil represent 5.1% of all the residues. For these residues the corresponding assignments by XTLSSTR, SEGNO and PROSS, are given.

**Figure 4:**
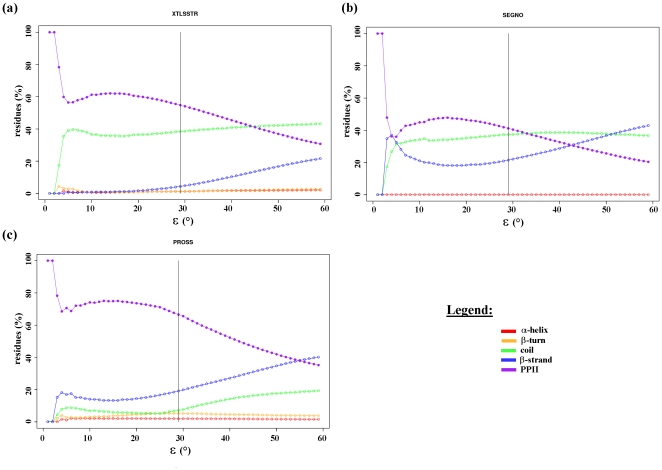
Secondary structure content in PPII assignments. The fig shows the percentage of residues of PPII^DSSP^ assigned as helix (red), β-turn (orange), coil (green), β-strand (blue) and PPII (purple) by (a) XTLSSTR, (b) SEGNO, and (c) PROSS, as a function of ε. The chosen ε (equals to 29°) is indicated by a black line.

Taking the suggestions by different groups into account [22], [37], [55], we have added a second rule to the PPII assignment approach: at least two consecutive residues with dihedral angles within the range (.φ+/−ε, .φ+/−ε), are required to form PPII helix. After an iterative search, a new ε of 29° was chosen for the assignment, which represents a PPII frequency of 5.11%. The average length of the PPII assigned within DSSP coil using this rule equals to 3.24 residues. The PPII assignment based on DSSP (named hereafter as PPII^DSSP^) corresponds to 56.0%, 45.6% and 65.9% of PPII assignments by XTLSSTR, SEGNO and PROSS respectively. This threshold ε has been selected by considering the following points: (i) the percent of amino acids assigned as PPII by one of the three SSAMs reached a maximum (of 83%), a further increase in ε decreased this percentage, and (ii) it is a good compromise between the average frequency of PPII observed (6.95%) and the average frequency of PPII within DSSP coil (4.48%). Mean φ and mean ψ values of PPII^DSSP^ are 75.3°and 141.1° respectively. Hence, this definition of PPII assignment is compared to the other PPII assignment methods and is consistent with theoretical definition of PPII. Table 1 summarizes the main results. Figure 3 gives the distribution of the different secondary structure states in the PPII^DSSP^ assignments corresponding to the chosen epsilon while Figure 4 shows the same information for the values of epsilon ranging from zero to 60°. While looking at XTLSSTR assignments corresponding to PPII^DSSP^ residues, the PPII content is generally high and mainly coil is found for low ε values. For SEGNO, which carries out least number of PPII assignments (see Figure 1), nearly 20% of the assignments correspond to β-strands. It must be noted that this SSAM does not assign any turns. Finally, PROSS shares the maximum number of PPII, the rest is mainly composed of β-strand and coil at a low percentage. The assignments are characterized by absence of helices and only a few turns were seen. Figure 4 shows the interest in considering a second consecutive dihedral angle (see Figure 2 to compare). Figure S4 gives the distribution of distance between extremities of PPII for different PPII lengths. A striking observation is the low standard deviation of the distance. It ranges between 1 and 2 Å depending on the length, while the deviation is 2–3 times higher for the PPII helices assigned by other SSAMs.

**Figure 5:**
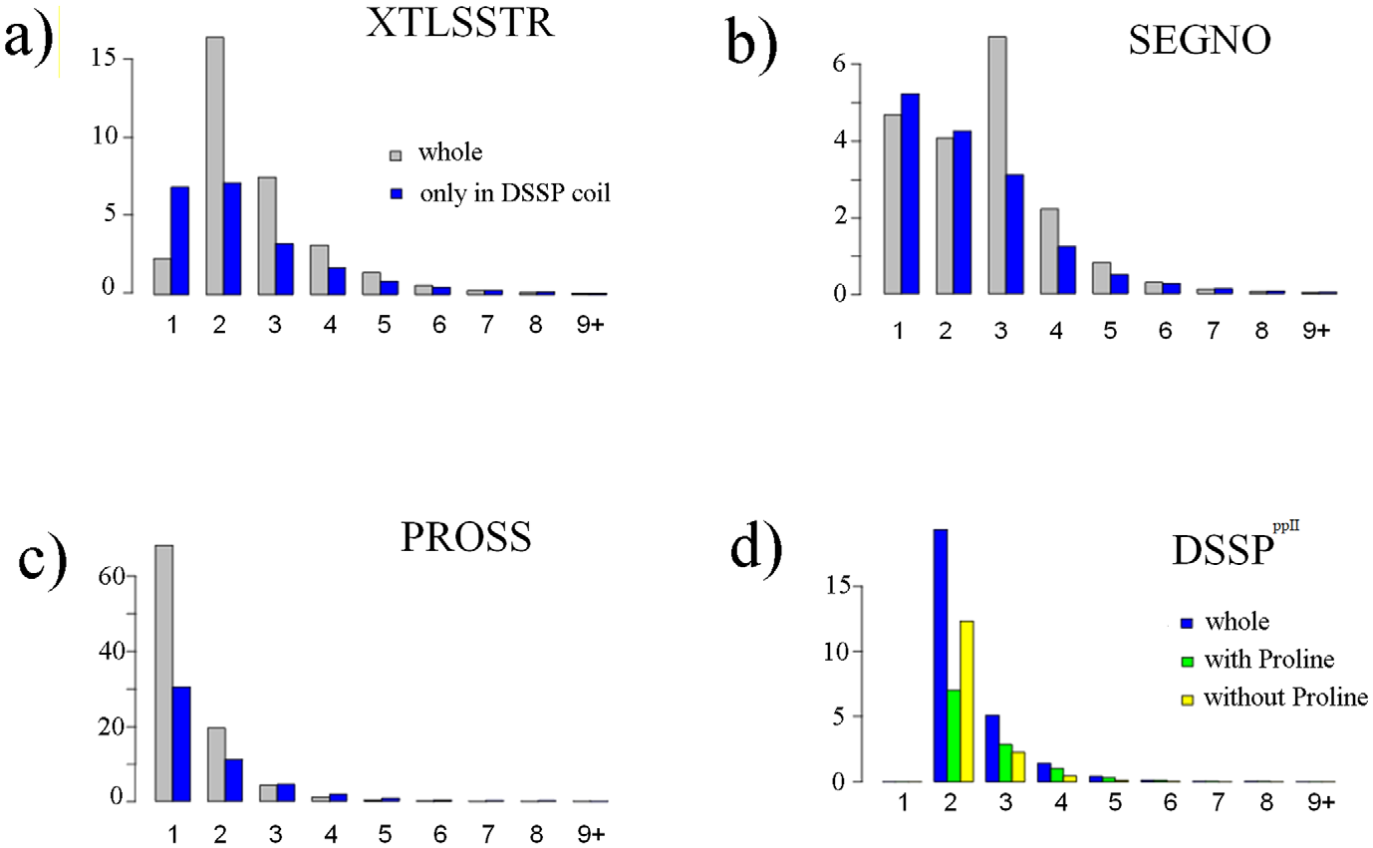
Length of PPIIs. For the three PPII assignment methods and the DSSP based assignment, the distribution across different lengths of PPII is given. The blue bars correspond to the PPII in coil assigned by DSSP, while the grey ones indicate the residues in all assigned states. For DSSP, the PPII assignments involving Proline (green) and those without any Proline (yellow) are also plotted.

As seen in Figure 5, for PROSS, this assignment criterion results in a small increase of the average length of PPII, from 1.35 to 1.63 amino acids (mainly due to a significant number of very short helices outside the DSSP coil). The average length in the case of SEGNO, decreases from 2.58 to 2.27 while for XTLSSTR, a decrease from 2.63 to 2.32 was observed.

#### Analysis of the sequence-structure relationship

**Figure 6:**
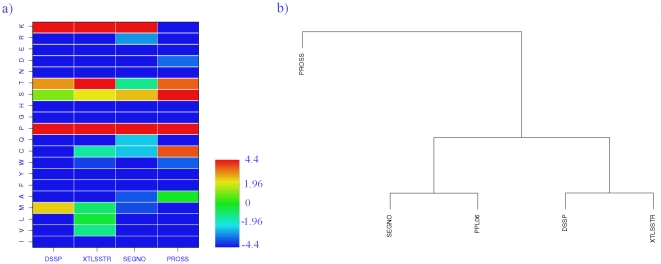
Amino acid distribution. (a) plot giving the Z-scores associated with the amino acids in the PPII assigned using DSSP (this study), XTLSSTR, SEGNO and PROSS. (b) Hierarchical clustering of the amino acid distribution frequencies associated with different assignments and the recent analysis done by Berisio and co-workers [37] (in terms of relative frequencies).

As expected, the amino acid preference for PPIIs differs depending on the SSAM used for assignment. However, the distribution of amino acids remains clearly different from that seen in other secondary structures (see Figure S5). Figure 6 summarizes the over and under-representation of amino acids in the PPIIs assigned by different SSAMs (including our new approach). All of them have about 3–5 over-represented amino acids. The most important one, as expected, is Proline (P) with a Z-score greater than 100. Then for all assignments, except PROSS (Z-score less than −10 and *p* less than 10^−5^), the other frequent amino acid is Lysine (K). The third important amino acid is Serine (S) which is seen more or less over-represented for the assignments by all the four SSAMs. It is highly preferred with respect to PROSS assignment (Z-score>4.4, *p*<10^−5^), considerable over-representation is found with SEGNO and XTLSSTR (Z-score>1.96, *p*<2.10^−3^) and in the case of our DSSP based assignment, occurrence frequency is similar to the background (Z-score equals to 1.1). Another important amino acid is Threonine (T), highly over-represented with XTLSSTR; strongly over-representation is seen with PROSS and DSSP, but slightly under-represented with SEGNO.

In some cases, the SSAM assignment results in assignment specific amino acid preferences. Significant preference for Methionine is found only with DSSP (Z-score equals to 2.44, *i.e. p*<10^−3^) while Cysteine (C) is seen strongly over-represented with PROSS (Z-score equals to 3.73, *i.e. p*<10^−3^). The hierarchical clustering based on the relative amino acid distribution frequencies of the four SSAMs and also the data from the work of Vitagliano's group [37], is shown on Figure 6b. DSSP and XTLSSTR show similar characteristics as in the case of SEGNO and [37]. PROSS gives the most distant distribution as it shares only 3 common over-represented amino acids (P, T and S), and has a unique over-representation of C.

**Figure 7:**
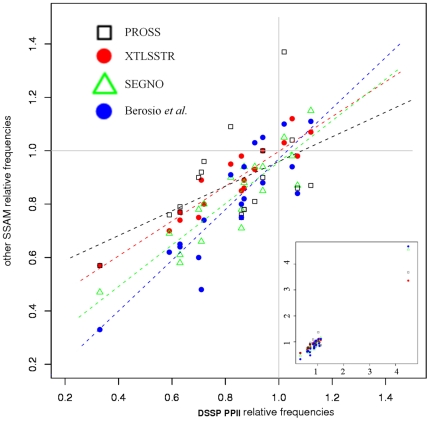
Amino acid relative frequencies. Plot of the relative frequencies of amino acids associated with PPII^DSSP^ with that of the PPII assigned by other SSAMs (red: XTLSSTR, green: SEGNO, black: PROSS) and the distribution obtained from the analysis of Berisio and co-workers [37](blue).

Figure 7 further highlights the characteristics of this distribution, giving the correlation of the relative preferences associated with PPII^DSSP^ with that of the other three SSAMs and also with the PPII analysis by Berisio *et al.* based on their assignment [37]. The correlation coefficients (excluding the Proline frequencies) are 0.89 with PROSS, 0.87 with the analysis of Berisio and co-workers, 0.82 with XTLSSTR, and only 0.53 with SEGNO. Between PROSS, XTLSSTR and the analysis of Berisio *et al.*, correlation coefficients range between 0.93 and 0.79. Including SEGNO reduces this further to about 0.59. This underlines (i) that PPII^DSSP^ is consistent with other PPII assignment methods and (ii) some significant differences can be seen considering one or the other assignment. The latter is important for the purpose of structure prediction and analysis, as PPIIs are the repetitive structures with the most contrasted residue distribution.

#### Analysis of amino acid preferences in PPII capping regions

**Figure 8:**
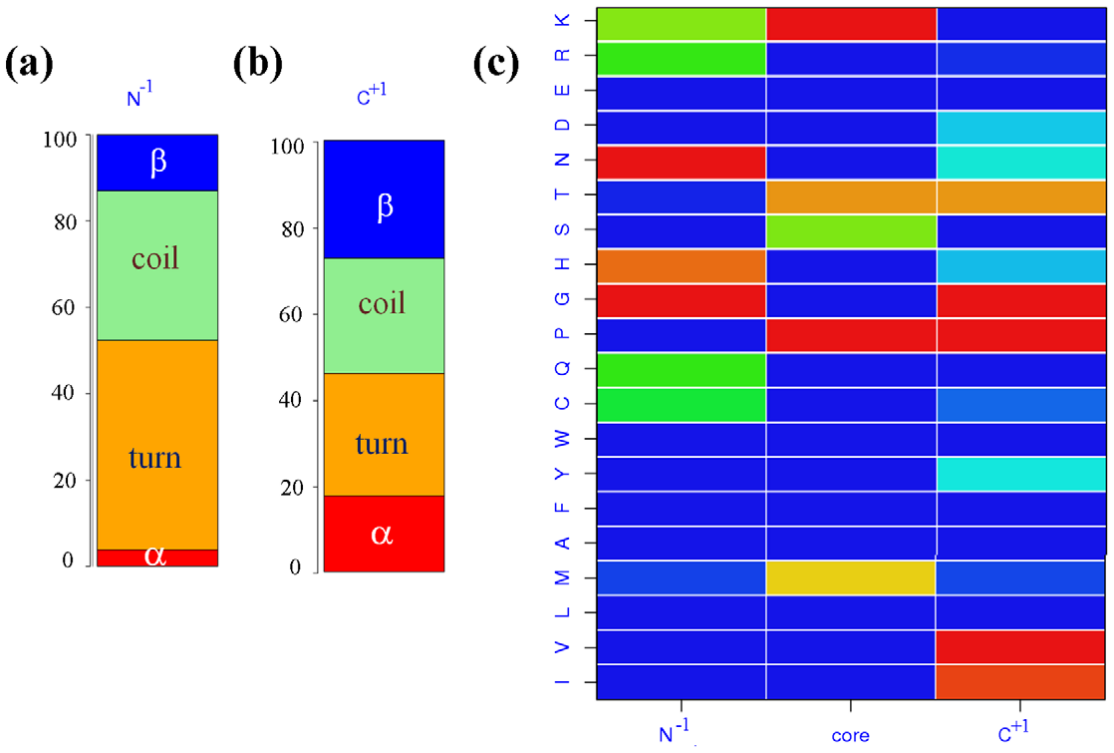
Sequence – structure relationship of PPII^DSSP^. The central upper part presents the plot of Z-scores associated with the amino acid distribution in PPII^DSSP^ and the capping regions (see Figure 3 for details). At its left and right the secondary structure distributions in these capping regions are shown (assigned by DSSP). The lower half of the figure shows the transitions of Protein Blocks in the Ncap to PPII^DSSP^ core (left) and from PPII^DSSP^ core to Ccap (right) . Only transitions with frequency more than 5% are shown.

Capping residues of PPII^DSSP^ (*i.e.*, amino acids before and after PPII^DSSP^) clearly have distinct amino acid preferences when compared to that of PPII^DSSP^ associated preferences (cf. previous paragraph). They are in fact close to coil and turn associated distributions. Figure 8 shows the amino acid distribution at the position just before the stretch assigned as PPII^DSSP^ (N^−1^) and the one after (C^+1^). N^−1^ has a high preference for Glycine (G) and Asparagine (N) and a considerable over-representation of Histidine (H), Glutamine (Q) and Lysine (K) is also seen. Histidine (H), Glutamine (Q) and Lysine (K) are also overrepresented in PPII. Lysine (K) has a Z-score of 1.2 in the N^−1^ and 7.0 within the PPII stretch. The distribution in the C^+1^ has more in common with that of PPII, characterized by high over-representations of G, P and V, strong over-representations of M and T and a significant representation of S.

Capping amino acids of PPII^DSSP^ are not only coil or β-sheet residues (see Figure S6). N^−1^ residues are mainly turn associated residues (with a frequency of 48.5% which is 2.4 times its expected frequency) and coil (34.7%, 1.8 times expected). β-sheet represents only 12.3% (0.55 times expected). For the C^+1^ residues, the frequencies are more equilibrated, still showing a considerable over-representation of turns (28.3%, 1.4 times its expected frequency).

#### Analysis of local structure features of PPII capping regions

To obtain a more detailed picture of the local structures associated with PPII, we have analyzed the capping regions in terms of a structural alphabet [56], [57], [60], *i.e.*, a set of small local protein structures that can be used to approximate precisely every part of a protein structure. Our structural alphabet, namely Protein Blocks [58], [59], [68], is composed of 16 distinct prototypes that are 5 residues long (see Methods section). It is the most widely used structural alphabet and has been proved useful in approaching several problems in the area of structural bioinformatics, *e.g.*, to compare protein structures [69], [70], [71], analyse sequence-structure specificities [72] or mine protein binding sites [73], [74].

This part of the study focuses on the precise analysis of the local protein conformations associated with PPII caps. Hence for the Ncap regions (see Figure S6, bottom left), within the PPII^DSSP^, PB *b* and PBs *i* to *p* (the latter set is largely associated with α-helices [75]), are never seen. To study the PB specificities in the cap regions, series of two PBs (di-PBs) were considered. Interestingly, 8 series of di-PBs correspond to 78% of all, seen in the Ncap regions of PPII (see Figure 8). These can be grouped into three main classes, the first one comprise 6 di-PBs and correspond to half of the Ncap associated di-PBs, the most important of these is *dd*. Many other strands related PBs are also found, which involves PBs *c* and *e*. The second most important series is *pa* (18.7%), *i.e.*, a series characterizing transition of α-helix to β-strand. The third one (8.2%) is *ia* which is largely associated with β-strand - β-strand transition [75]. Hence, different neighbourhoods are observed.

The Ccap is more conserved with only 6 di-PBs corresponding to 87% of the observations. Two main behaviours were observed. A first cluster is associated with longer PPII^DSSP^s, involving series *fk* (25.8%), *fb* (11.5%) and *hi* (11.6%), while shorter PPII^DSSP^ are still strongly linked to beta-like PBs with series *dd* (19.1%), *cd* (13.8%) and *df* (6.0%). Thus, PPII^DSSP^ have a strong local signature depending on the neighbourhood and more complex than expected.

#### Analysis of structural properties

**Figure 9:**
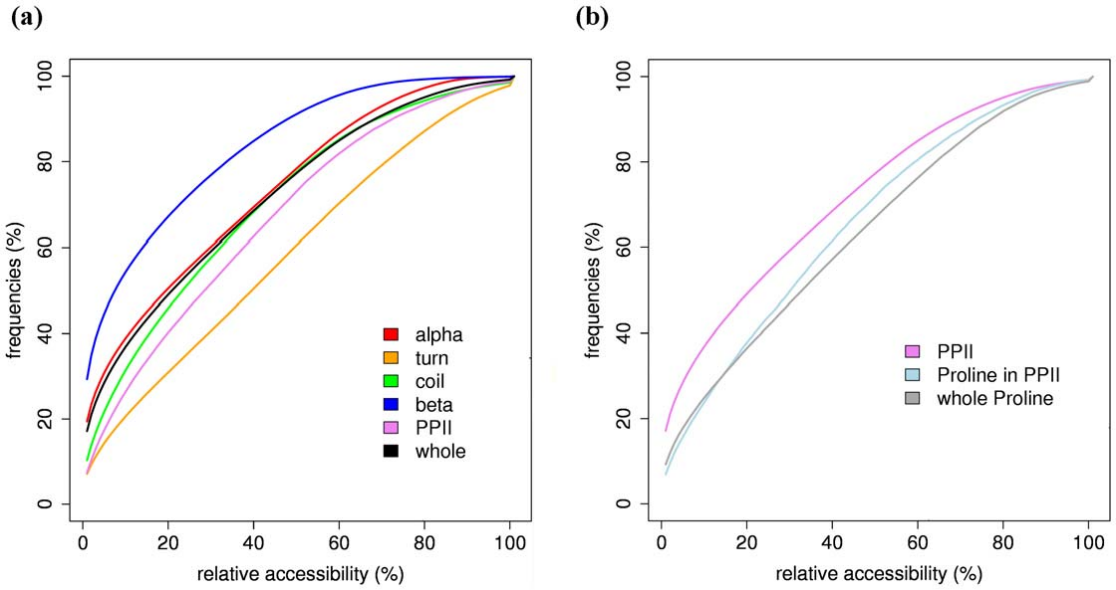
PPII^DSSP^ accessibility. Plots showing the relative accessibility of the different secondary structures (left), and the relative accessibility ofPPII^DSSP^ (with and without proline residues) and that of all Prolines.

PPII are considered as potential interacting regions, hence an analysis of their solvent accessibility will be of broad interest. Relative accessibility of different secondary structures is presented in Figure 9a. PPII^DSSP^ is the second most accessible secondary structure, following the turns and hence they are quite different from β-strands which are the less accessible (on an average). For a relative accessibility threshold of 25%, only 46.1% of PPII^DSSP^ are buried while in the case of turns, β-strands, α-helices and coils, 35.8%, 72.2%, 55.4% and 51.9% of residues are buried, respectively. Thus, PPII^DSSP^ is more accessible than coils also. Interestingly, PPII^DSSP^ with Proline are more accessible than PPII^DSSP^ without Proline (see Figure 9b). Accessibility of Proline associated with PPII^DSSP^ is high and does not really differ from the average accessibility of Proline.

The average numbers of contacts have been analyzed, using a classical distance based approach, *i.e.*, a contact is defined if a distance between atoms is less than 8.0 Å [76], [77], [78]. Unlike the accessibility, PPII^DSSP^ is similar to β-strand in terms of the average number of contacts. The turn and coil have lesser contacts and the contacts are more in the case of α-helices. These results are in accordance with previous studies on PPII^DSSP^ reflecting the relevance of the rules used to define it.

#### A case of molecular modeling

**Figure 10:**
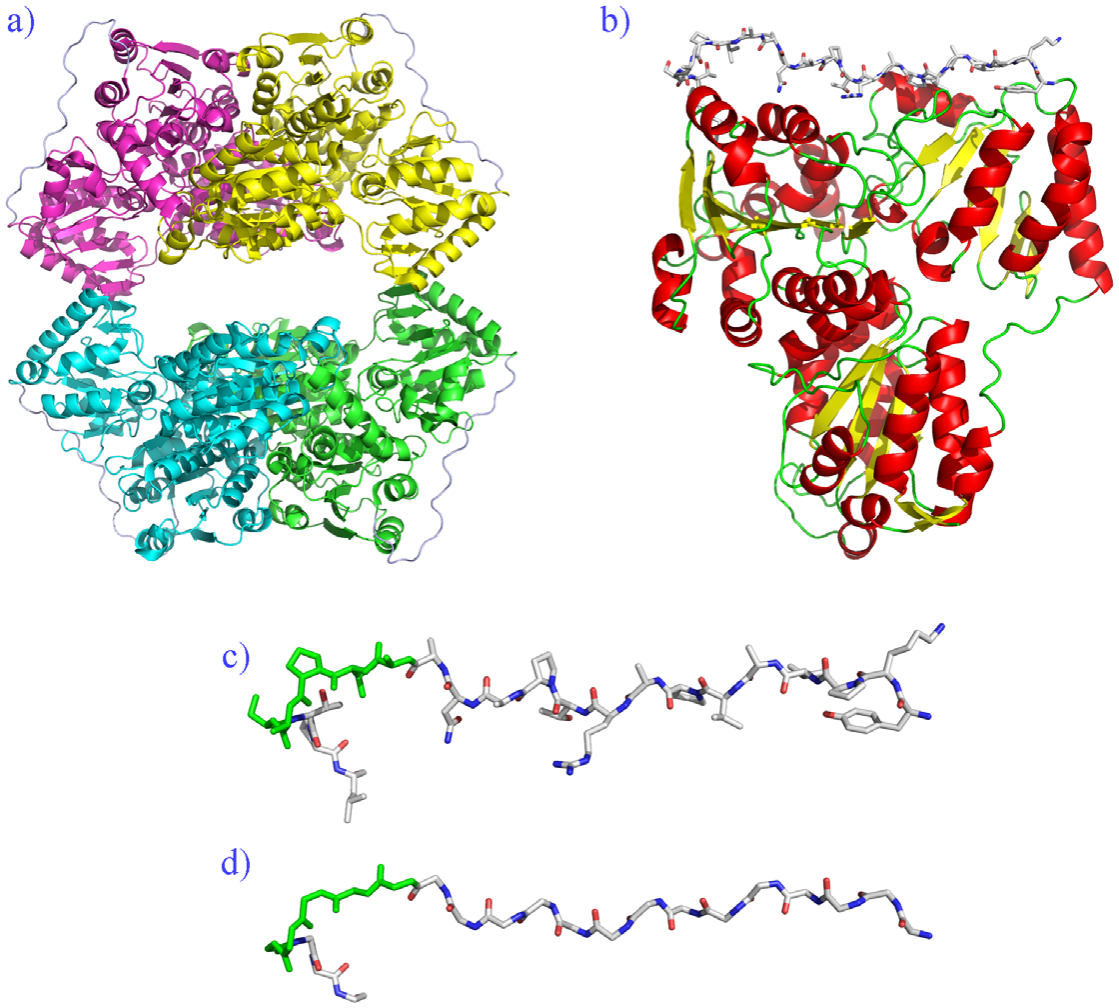
Saccharomyces cerevisiae pyruvate decarboxylase. (a) The structure involving four chains (PDB code 2VK8 [80]). (b) Chain A represented in cartoon, with a long loop associated with PPII conformation, shown in stick representation. The long loop with both backbone and sidechain atoms (c) and with only only backbone. The connecting non PPII^DSSP^ region is shown in green.

**Figure 11:**
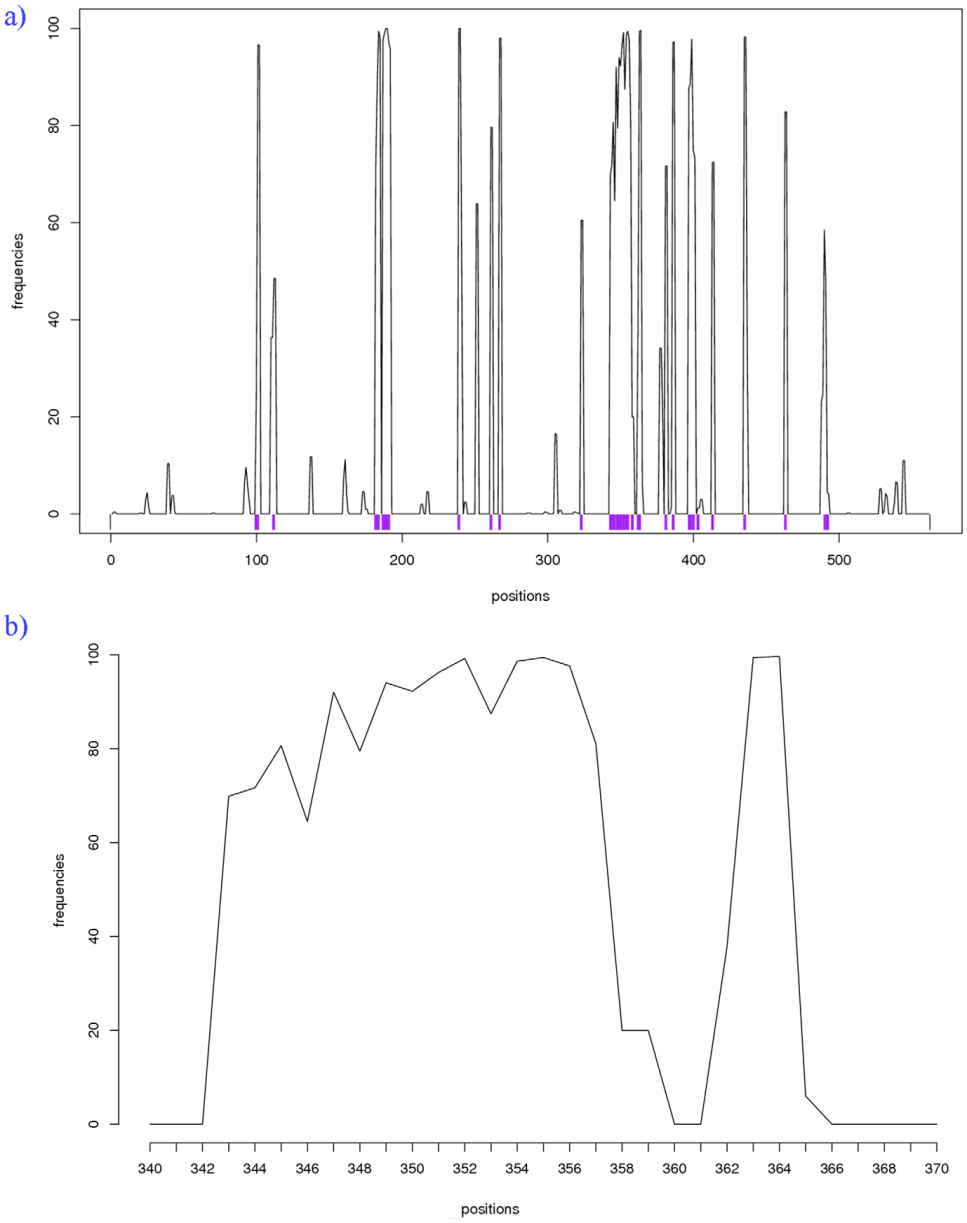
Residues assigned as PPII^DSSP^. Frequencies of PPII^DSSP^ assignments during course of MD simulation shown for (a) the whole protein and (b) the PPII associated long loop (see Figure 10). The positions initially assigned as PPII^DSSP^ are shown in purple.

Finally, to study the dynamic behaviour of PPII (assigned using our approach), we carried out a Molecular Dynamics simulation. Molecular dynamics force field parameters seem to underestimate the polyproline II and thus diminish their frequencies [79]. For this purpose, we selected *Saccharomyces cerevisiae* pyruvate decarboxylase (PDB code 2VK8 [80]) which has a high PPII^DSSP^ content, about 12% (see Figures 10a and 10b) and one of these PPII helices is quite long (see Figures 10c and 10d). The simulation has been carried out using GROMACS 4.0.5 [81], [82], [83], [84] with the OPLS-AA force field [85], details can be found in Figures S7 and S8. Figure 11a gives the frequency of PPII^DSSP^ assignment for each residue during the simulation. The majority of residues initially associated to PPII^DSSP^ stays associated to this state; only 17% of these residues have a frequency of PPII^DSSP^ less than 50%. Even, some residues initially not associated to PPII^DSSP^ state, becomes associated to this conformation during the course of the simulation. It is striking that the long PPII helix is in fact, 2 residues longer than initial. The evolution of the relative frequency of PPII^DSSP^ during the simulation shows only a mean loss of 11% of PPII^DSSP^ content. This value is not dependant on the time of the simulation and more interestingly, is equivalent to the mean loss of other repetitive structures (see Figure 11b). These results suggest a better conservation of PPII than previously observed in molecular dynamics simulations [33], [86]. This can be explained by the fact that the previous studies mainly focus on PPII fragments and not the PPII content with a protein structure. This was also highlighted in the work of Zagrovic and co-workers [79]. Indeed, recent studies have shown the crucial impact of related force-fields on PPII conformation and the beta-strands contents, which also seem to be associated. AMBER-03 significantly overweighs the contribution of extended and PPII backbone configurations to the conformational equilibrium while AMBER-99SB variant shows a strong bias towards extended beta and PPII conformations [87].

## Discussion

A long history of experimental analyses of peptides with PPII conformation exists. This involved the study of chemical activities under different conditions [45], [88], [89]. However, in the field of structural bioinformatics, PPII has been a subject of only a limited number of studies. The majority of studies on PPII concern protein folding [90], while few have focused on model building and sequence-structure relationships [91].

Pro-rich sequences are common recognition sites for protein–protein interaction , *e.g.*, the SH3 domain or the WW domain [92]. Hence protein – protein interaction involving PPII is also an important area of interest [93], [94], [95]. We can note for instance, the protein PflI which is a protein involved in flagellar positioning in *Caulobacter crescentus* possess a PPII helix, implicated in interactions [96]. Rath, Davidson and Deber concluded a crucial review on PPII with these sentences: “An increasing amount of evidence suggests that so-called “random-coil” polypeptides may not have completely irregular structure, but are more accurately described largely as PPII helices. This observation, along with the importance of PPII structure in protein– protein recognition elements, implies that the Polyproline II conformation should be regarded as equally important to the folding and function of proteins as the classical α-helix, β-sheet, and β-turn structures.” [97].

As observed in many studies, local structure assignment is not trivial [56]. As these assignment methods are based on various parameters and definitions for repetitive structures, they often give different assignments [63], [67], [68], [98]. PPII is an important local protein structure, but not given enough significance, as noted by Deber [97] and Rose [19]. Here we propose a simple strategy to assign PPII on the basis of the most widely used SSAM, *i.e.*, DSSP. For this purpose, two major points must be considered. The first is to define a rule that is largely coherent with the available PPII assignment methods. The second is to assess the sequence – structure relationship and check if the results are in agreement with the literature. It would emphasize the quality of our PPII assignment.

At first, we observe that confusions between the SSAMs (DSSP, XTLSSTR, SEGNO and PROSS [21], [53], [54], [55]) used in this study correlate well within the classical *C*_3_ values of about 80% [51], [56], [63], [98].No strange behaviours (*e.g.*, DEFINE [99] that has a *C*_3_ value close to 60% [51]) were observed.

The analysis of the relative frequency of PPII reveals a complex issue. As mentioned earlier, a ratio of 2.5 is observed while comparing the frequencies of occurrence of PPII, based on the assignments by SEGNO and PROSS, with the occurrence frequencies ranging from 4.0 to 10.1%. The results of works done by other groups also highlight this issue. Adzhubei and Sternberg found them more common than expected [91]. Jha and co-workers even ascribed a singular dominance to PPII based on their coil library [34]. However, the work of Daggett's group did not confirm this [100] while Berisio and co-workers found an occurrence rate of 2% [37].

Another issue is the way turns are treated. In many works on the analysis of secondary structures, assignment of turns has not been considered. For instance, SEGNO assigns helices, sheets and PPII, but not the turns. In a same way, Berisio and co-workers used PROMOTIF to assign α- and 3_10_-helices and β-strands, and then assign PPII based on their own rules [37]. As turns are important and cannot be neglected [65], we have decided to consider them prior to the PPII assignment.

Using simple rules based on the choice of a set of (φ, ψ) dihedral angles for assigning PPII^DSSP^, led to a good agreement with the assignment made by other SSAMs. After different tests, we have selected a range of +/−29° around the canonical values of PPII. This gives a PPII occurrence frequency of 5.1%, representing about 1/3^rd^ of the coil state and leading to an average length of 3.2 residues per PPII^DSSP^ helix. This assignment is coherent with distribution of PPII based on the assignments made by other SSAMs (see Table 1 for a summary).

Interestingly, our results show a good concordance with recent studies and also the assignment done by the other SSAMs. It must be noted that the agreement was poor when compared to the earlier studies with fewer data [22], [24], [101], the correlation coefficients were between 0.1 and 0.3. Moreover, the amino acid preferences of PPII observed, is similar to that seen in the assignments made by other SSAMs and published studies. This is characterized by a strong over-representation of P (as expected), K, and a considerable preference for M, T and S. Since long, there has been evidence for the presence of PPII conformations in non-proline polypeptides [26], [102], we observe this predominantly in the shorter helices (see Figure 2).

PPII helices have been implicated in protein–protein recognition and folding. PPII conformation in stabilized in the unfolded polypeptides [97] and polymers of proline in aqueous solution are known to adopt this conformation as a result of steric interactions between prolyl rings [23]. Several studies report that PPII helices are more surface exposed than other repetitive structures [22], [24], our results agree entirely with these findings. Interestingly, its accessibility is not as high as turns which are more accessible than any other secondary structures, including coils. Analysis of capping regions of PPII shows pertinent properties. Especially the Ncap could be roughly characterized as a ‘turn’ without Proline. Recent studies have also highlighted the difference in the type of interactions between secondary structures. n→π* interactions favour contacts between α–helix and PPII while dipole–dipole interactions are frequent between β–sheet and PPII and long-range backbone H-bonds bridge α–helix and β–sheet conformations [103], [104].

In conclusion, it can be seen that though our approach is coarse and simple, it presents considerable insights into the understanding of PPII. The results are in good agreement with that of the earlier studies on PPII. Moreover, the PPII^DSSP^ helices are longer than PPII helices of the other SSAMs. Implementation of such an approach is quite easy. However, one must note that an *a posteriori* assignment is perhaps not the optimal assignment. The choice of DSSP is mainly due to its popularity though other methods exists which are quite efficient, *e.g.*, STRIDE [105]. Assignment made by STRIDE has 95% agreement with DSSP [51]. Satisfactorily, using our approach, 96.7% of PPII^STRIDE^ is also assigned as PPII^DSSP^. Like SEGNO, the assignment rules could be adapted to give different assignments for the core and extremities of PPII [36]. Nonetheless, our approach could assist in highlighting the importance of PPII as a repetitive structure and widening the extent of research carried out on PPII [97].

## Materials and Methods

### Data sets

The dataset of protein structures is taken from the PISCES database [61], [62] and represents 1,732,996 amino acids from 6,665 proteins chosen based on a pairwise sequence identity cutoff of 30% with resolution less than 2.5 Å and R factor below 0.2. It is available at http://www.dsimb.inserm.fr/~debrevern/DOWN/DB/PPII. Each chain is complete and does not have missing residues [51], [68].

### Secondary structure assignment

Assignment has been carried out with four different methods: DSSP [21] (CMBI version 2000), XTLSSTR [53], PROSS [54] (version September 2004) and SEGNO [55] (version 3.1). DSSP, PROSS, XTLSSTR and SEGNO assign more than five secondary structural states, thus we have reduced them as: α-helix includes α, 3._10_ and α- helices, the β-strand contains only the β-sheet, the turn involves the turn assignments and bends (which are assigned by DSSP), the PPII corresponds to the PolyProline II assignments (not assigned by DSSP) and the coil includes the rest of the assignments (β-bridges and coil). Default settings have been used for all methods.

### Protein Blocks description

Protein Blocks (PBs [60]) correspond to a set of 16 local prototypes, labeled from *a* to *p* (see Figure 1 of [68]), of 5 residues length, clustered based on φ, ψ dihedral angles description. They were obtained using an unsupervised classifier similar to Kohonen Maps [106] and Hidden Markov Models [107]. The PBs *m* and *d* can be roughly described as prototypes for central α-helix and central β-strand, respectively. PBs *a* through *c* primarily represent the N-cap region of β-strand while PBs *e* and *f* correspond to the C-caps; PBs *g* through *j* are specific to coils, *k* and *l* correspond to the N cap region of α-helix, and PBs *n* through *p* to that of C-caps. This structural alphabet allows a reasonable approximation of local protein 3D structures [59] with an average root mean square deviation (*rmsd*) of 0.42 Å [58]. PB [58] assignment was carried out using an in-house program written in C (available at http://www.dsimb.inserm.fr/~debrevern/DOWN/LECT/), it follows similar rules to assignment done by PBE web server (http://bioinformatics.univ-reunion.fr/PBE/) [71].

### Agreement rate

To compare two distinct secondary structure assignment methods, we used an agreement rate *C*_3_, which is the proportion of residues associated to the same secondary structure state [63]. Note that SEGNO does not assign turns.

### Z-score

The amino acid occurrences for each secondary structure have been normalized to a Z-score [59], [108], [109], [110]:
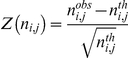



where 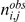 is the observed occurrence number of amino acid *i* in position *j* for a given secondary structure and 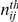 the expected number. The expected frequency is given by the product of the occurrences in position *j* with the frequency of occurrence of amino acid *i* in the entire databank. Positive Z-scores correspond to overrepresented amino acids and respectively negative z-score for underrepresented; threshold values of 4.42 and 1.96 were chosen (probability less than 10^−5^ and 5.10^−2^ respectively) to assess the significance.

## Supporting Information

Figure S1 **Agreement rate of SSAMs (reduced to three-states).**
(DOC)

Figure S2 **Secondary structure frequencies of the different SSAMs.**
(DOC)

Figure S3 **Ramachandran maps.** a) full databank, PPII assigned by b) PROSS, c) XTLSSTR and d) SEGNO.
(DOC)

Figure S4 **Distance between extremities of PPII assigned through coil DSSP.**
(DOC)

Figure S5 **Clustering based on the amino acid distribution in the assignments made by different SSAMs.**
(DOC)

Figure S6 **PPII capping regions.**
(DOC)

Figure S7 **Molecular dynamics of Saccharomyces cerevisiae pyruvate decarboxylase (PDB code 2VK8).**
(DOC)

Figure S8 **Molecular dynamics of Saccharomyces cerevisiae pyruvate decarboxylase (PDB code 2VK8).** [animation]
(DOC)
