# Supplementary material for: Assignment of PolyProline II Conformation and Analysis of Sequence – Structure Relationship
Source: PLoS One. 2011 Mar 31;6(3):e18401. doi: 10.1371/journal.pone.0018401 (PMC3069088; doi:10.1371/journal.pone.0018401)
Supplement: Figure S1 — Agreement rate of SSAMs (reduced to three-states). (DOC) [file pone.0018401.s001.doc]

**Figure S1.** *Agreement rate of SSAMs (reduced to three-states).*

|  | XTLSSTR | SEGNO | PROSS |
| --- | --- | --- | --- |
| DSSP | 77.4 | 88.9 | 82.3 |
| XTLSSTR | -- | 73.5 | 73.9 |
| SEGNO |  | -- | 82.4 |
| PROSS |  |  | -- |

**Table of *C*_3_ values.**


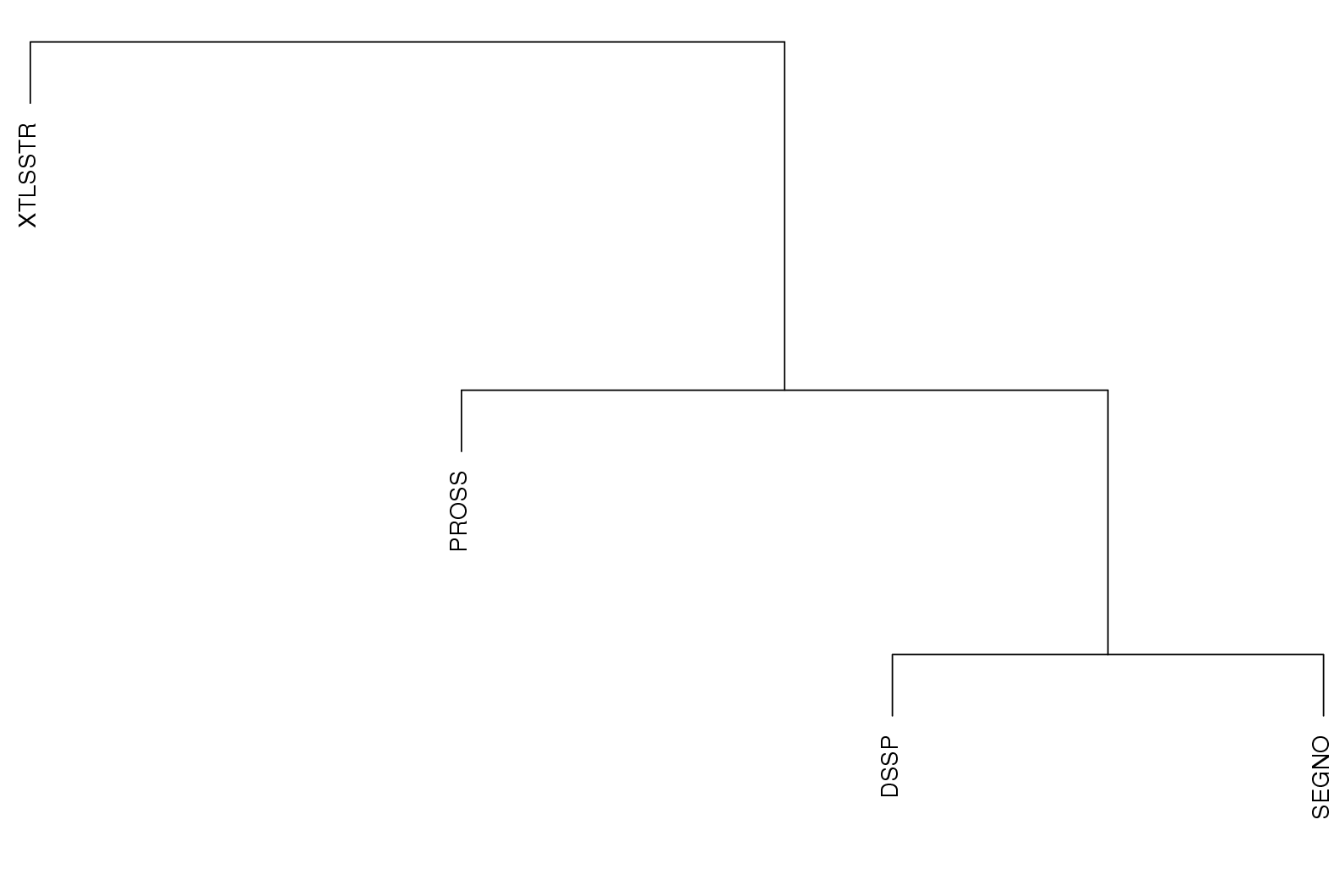


**Hierarchical clustering of *C*_3_ values** (done with R software)**.**

It underlines the proximity of DSSP and SEGNO which shares the highest C3 while XTLSSTR remains the most different SSAM.
