# Supplementary material for: Assignment of PolyProline II Conformation and Analysis of Sequence – Structure Relationship
Source: PLoS One. 2011 Mar 31;6(3):e18401. doi: 10.1371/journal.pone.0018401 (PMC3069088; doi:10.1371/journal.pone.0018401)
Supplement: Figure S2 — Secondary structure frequencies of the different SSAMs. (DOC) [file pone.0018401.s002.doc]

**Figure S2.** *Secondary structure frequencies of the different SSAMs*.

|  | DSSP | XTLSSTR | SEGNO | PROSS |
| --- | --- | --- | --- | --- |
| α-helix | 38.2 | 27.8 | 37.2 | 34.5 |
| turn | 19.8 | 7.8 | -- | 13.9 |
| coil | 19.8 | 44.1 | 35.9 | 21.3 |
| β-sheet | 22.2 | 13.5 | 22.9 | 20.2 |
| PPII | -- | 6.8 | 4.0 | 10.1 |
