# Supplementary material for: Assignment of PolyProline II Conformation and Analysis of Sequence – Structure Relationship
Source: PLoS One. 2011 Mar 31;6(3):e18401. doi: 10.1371/journal.pone.0018401 (PMC3069088; doi:10.1371/journal.pone.0018401)

**Supplementary Data 3.** *Ramachandran maps.* a) full databank, PPII assigned by b) PROSS, c) XTLSSTR and d) SEGNO.


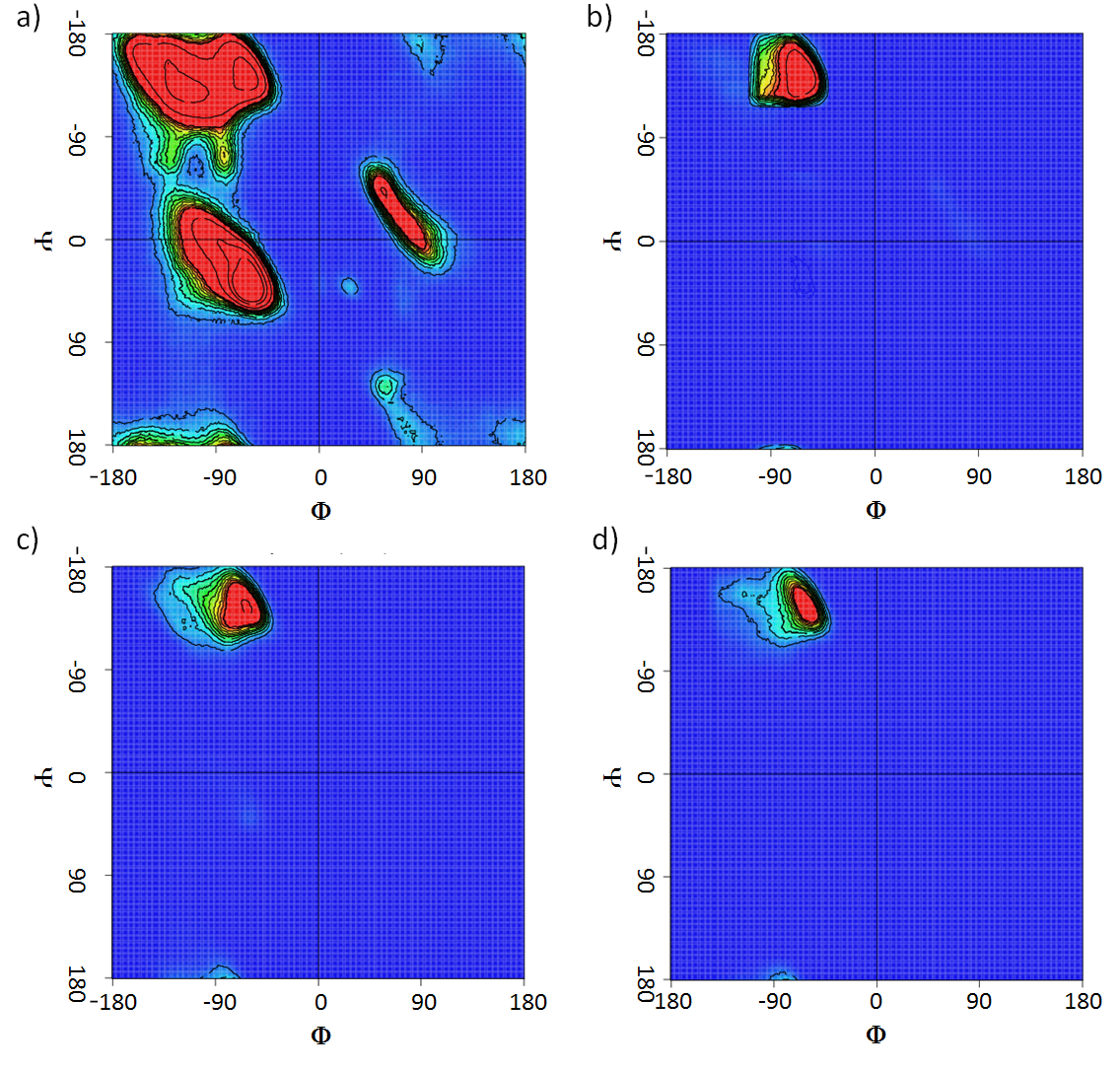

Supplement: Figure S3 — Ramachandran maps. a) full databank, PPII assigned by b) PROSS, c) XTLSSTR and d) SEGNO. (DOC) [file pone.0018401.s003.doc]
