# Supplementary material for: Assignment of PolyProline II Conformation and Analysis of Sequence – Structure Relationship
Source: PLoS One. 2011 Mar 31;6(3):e18401. doi: 10.1371/journal.pone.0018401 (PMC3069088; doi:10.1371/journal.pone.0018401)
Supplement: Figure S4 — Distance between extremities of PPII assigned through coil DSSP. (DOC) [file pone.0018401.s004.doc]

**Figure S4.** *Distance between extremities of PPII assigned through coil DSSP*.


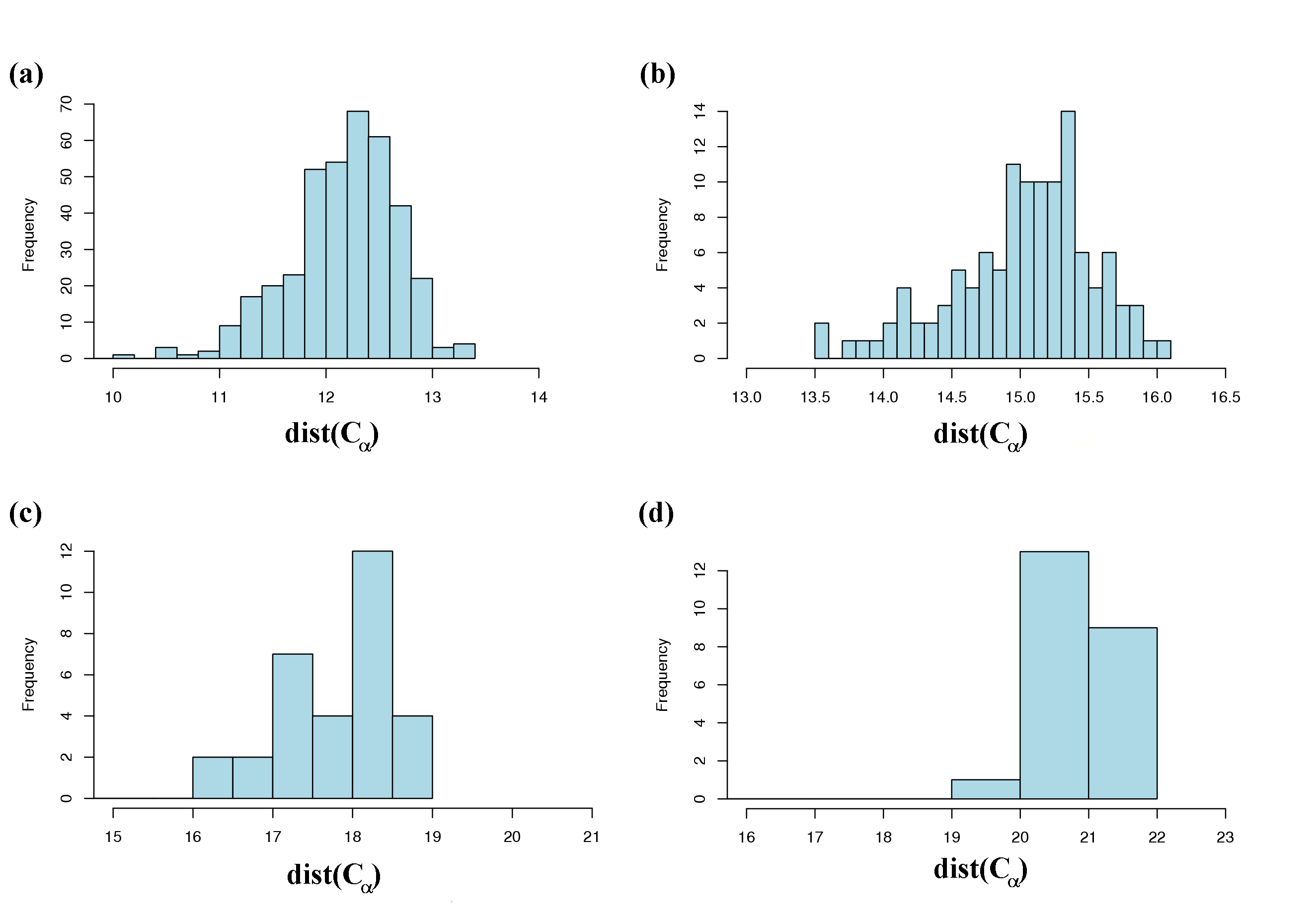


Are given the distances of PPIIDSSP ranging from 5 (a) to 8 (d) residues. The standard deviations are ranging from 0.7 to 1.7 A while they ranged from 2.9 to 4.5 for SEGNO and 2.7 to 5.5 for PROSS.
