# Supplementary material for: Assignment of PolyProline II Conformation and Analysis of Sequence – Structure Relationship
Source: PLoS One. 2011 Mar 31;6(3):e18401. doi: 10.1371/journal.pone.0018401 (PMC3069088; doi:10.1371/journal.pone.0018401)

**Figure S5.** *Clustering based on the amino acid distribution in the assignments made by different SSAMs*.


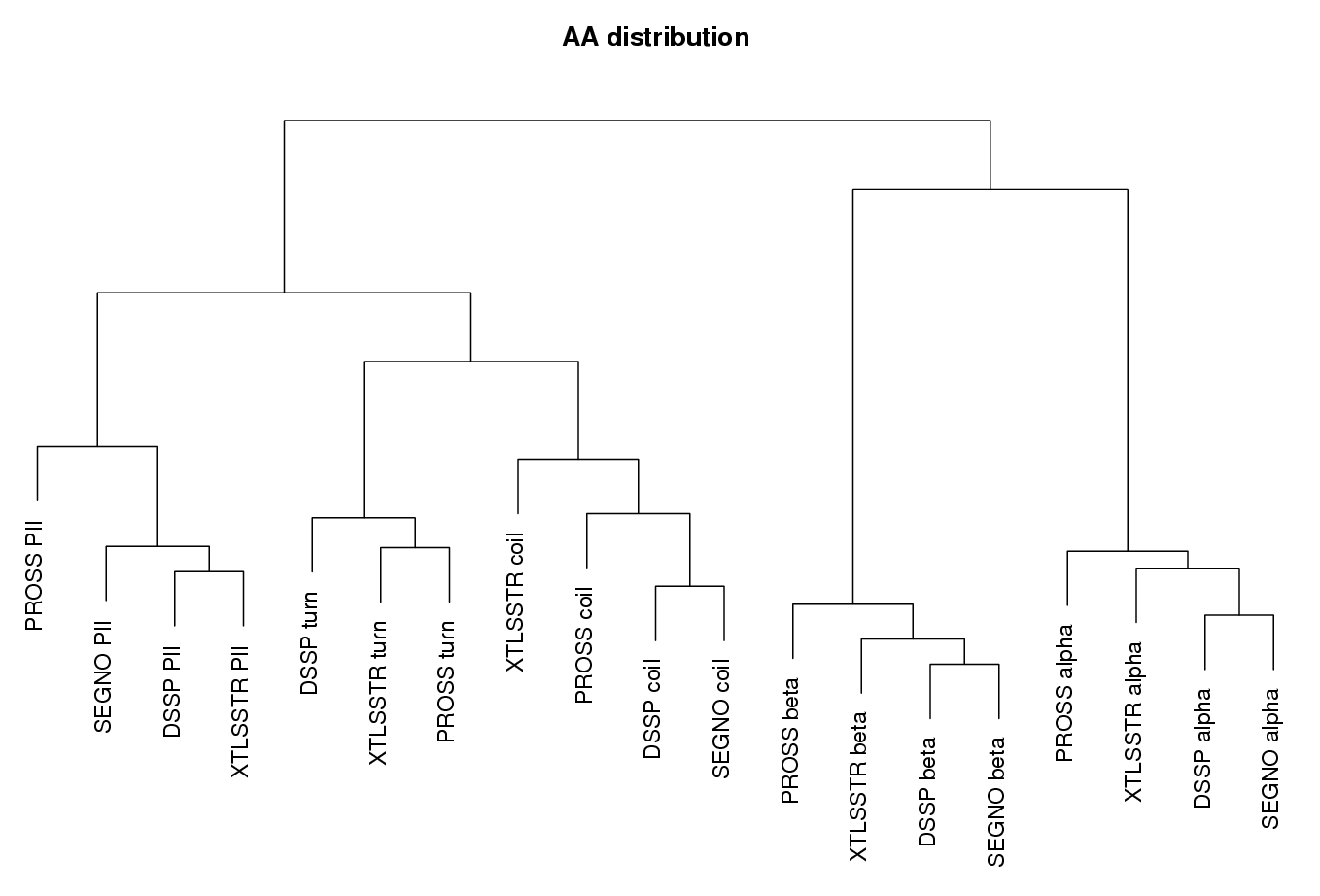

Supplement: Figure S5 — Clustering based on the amino acid distribution in the assignments made by different SSAMs. (DOC) [file pone.0018401.s005.doc]
