# Supplementary figures and images for: Assignment of PolyProline II Conformation and Analysis of Sequence – Structure Relationship
Source: PLoS One. 2011 Mar 31;6(3):e18401. doi: 10.1371/journal.pone.0018401 (PMC3069088; doi:10.1371/journal.pone.0018401)

**Figure S6.** *PPII capping regions*.


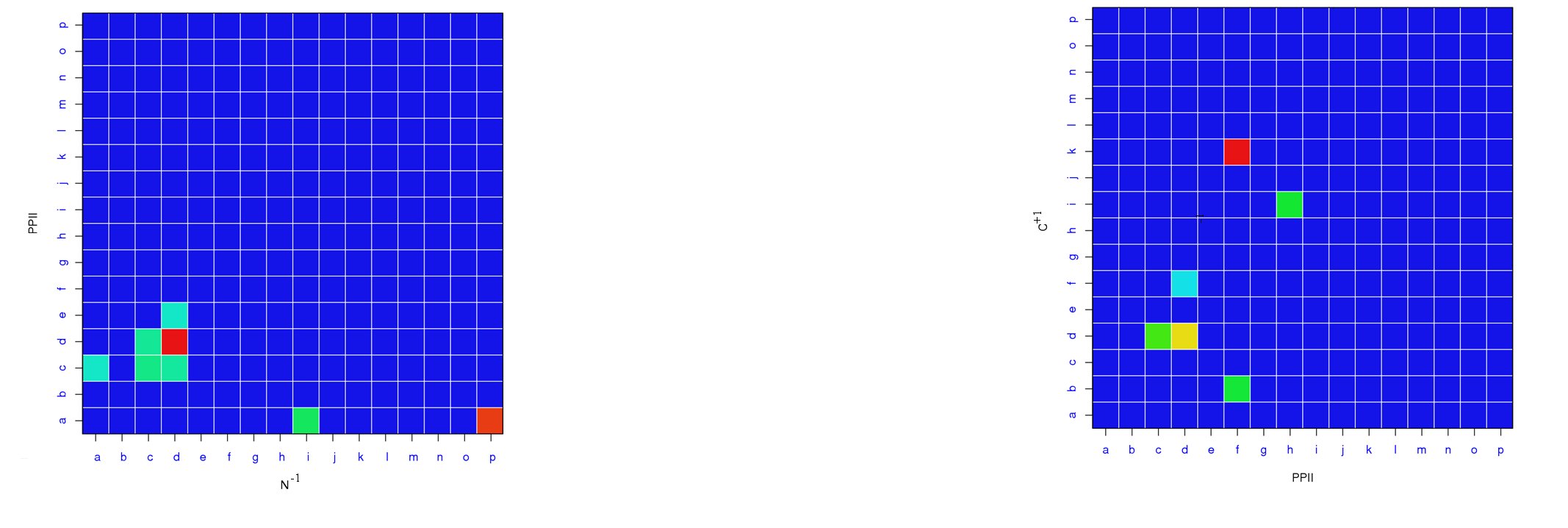


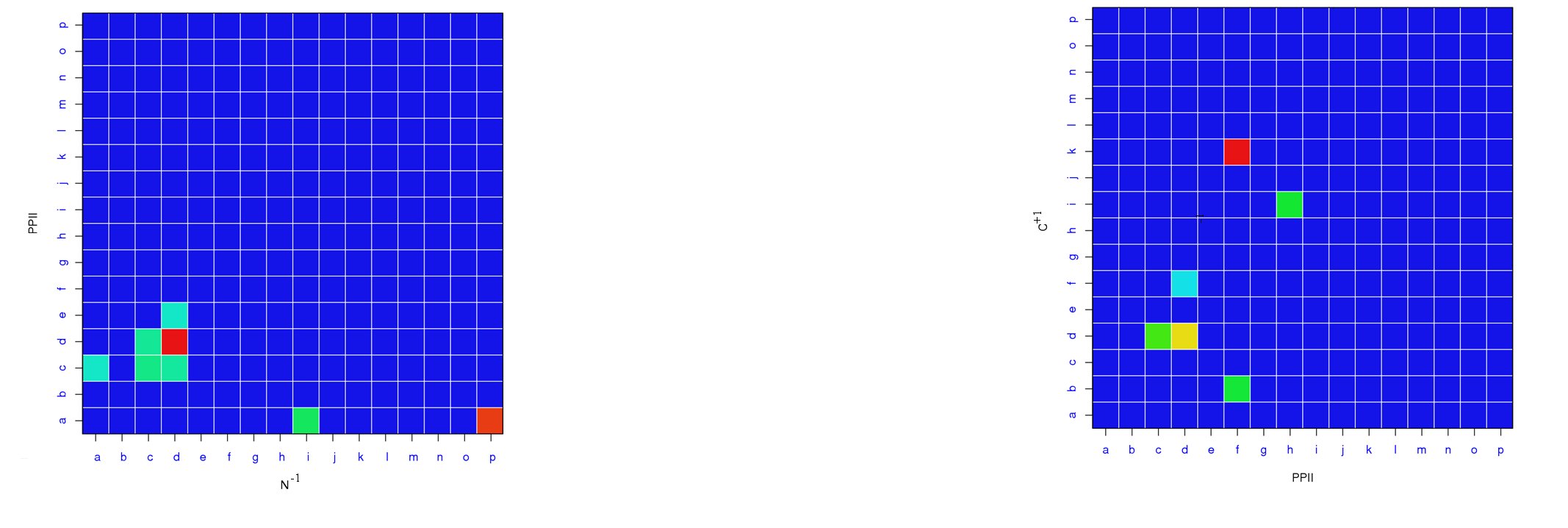

Supplement: Figure S6 — PPII capping regions. (DOC) [file pone.0018401.s006.doc]
