# Supplementary material for: Assignment of PolyProline II Conformation and Analysis of Sequence – Structure Relationship
Source: PLoS One. 2011 Mar 31;6(3):e18401. doi: 10.1371/journal.pone.0018401 (PMC3069088; doi:10.1371/journal.pone.0018401)
Supplement: Figure S7 — Molecular dynamics of Saccharomyces cerevisiae pyruvate decarboxylase (PDB code 2VK8). (DOC) [file pone.0018401.s007.doc]

**Figure S7.** *Molecular dynamics of Saccharomyces cerevisiae pyruvate decarboxylase (PDB code 2VK8)*.

We have selected one subunit of the yeast pyruvate decarboxylase deposited by Kutter and co-workers (Kutter et al. 2009) in the Protein DataBank (Berman et al. 2000). We have chosen chain ‘A’ (see Figure 11b); the 4 chains were identical (see Figure 11a) and solved with high- resolution (1.42 Å). This protein contains one of the longest chains of PPII^DSSP^ observed in our non-redundant databank. This loop (see Figure 11c and 11d) extends from residues 345 to 357, the smallest PPII helix traverse positions 362-364. Other SSAMs also assigned PPII at these positions (King and Johnson 1999; Srinivasan and Rose 1999; Cubellis et al. 2005).

Molecular dynamics (MD) simulations were performed with GROMACS 4.0.5 software (Lindahl et al. 2001; Berendsen et al.; van der Spoel et al. 2005; Hess et al. 2008) using OPLS-AA force field (Jorgensen et al. 1996) for proteins and the TIP 4P model for water was used (Jorgensen et al. 1983). The structure was then immersed in a periodic water box neutralized with Na^+^ or Cl^-^ counterions. Each system was then energy-minimized with a steepest-descent algorithm for 1000 steps. Once the system was heated at 300K, MD simulation was performed in NPT ensemble, with temperature and pressure kept constant at 300 K and 1 bar respectively using Berendsen algorithm (Berendsen et al. 1984). The coupling time constants were τ=0.1 ps and τ=0.5 ps for temperature and pressure respectively. Bond lengths were constrained with the LINCS algorithm (Hess et al. 1997), which allowed an integration step of 2fs. The generalized reaction field algorithm (Tironi et al. 1995) was used for long-range electrostatic interactions using a dielectric constant of 54 and a cut-off of 1.4 nm for non-bonded interactions.

A MD simulation was first perforned for 100 ps, with protein atom positions constrained while ions and water molecules were free. Then the simulation was fully relaxed and equilibrated for 5 ns. The coordinates were recorded at every ps interval (see Supplementary data 8a).

The MD was checked and analyzed using Gromacs tools. For instance, Figure S7.1 shows the variation of Cα root mean square deviation (RMSD), as a function of time. After 600 ps, RMSD reached a plateau of 1.5 Å with respect to the initial conformation at the beginning of the simulation.


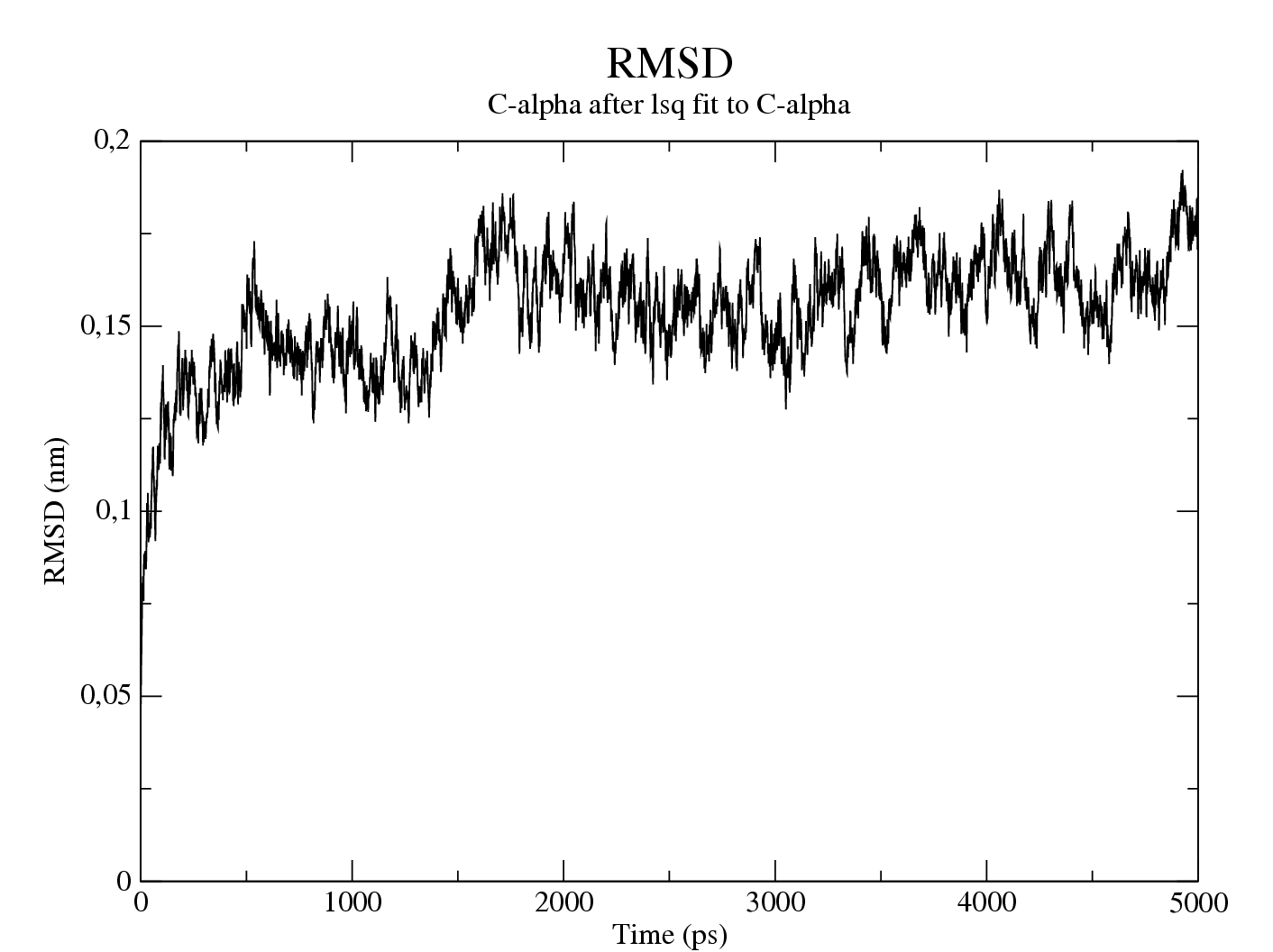


**Figure S7.1.** Variation of Cα RMSd .as a function of time

PII^DSSP^ frequency was computed during the simulations. PII^DSSP^ frequency was quite high, *i*.*e*., 12 %, more than twice the classical observed frequency. Figure S7.2 shows the variation of relative frequency of PPII^DSSP^ during the simulation, when compared to the initial PPII ^DSSP^ content (100%), An average conservation of 88.8% or only a mean loss of 11% is observed. This value does not depend on the duration of the simulation and, more interestingly is similar to the loss in the frequency of other repetitive structures.


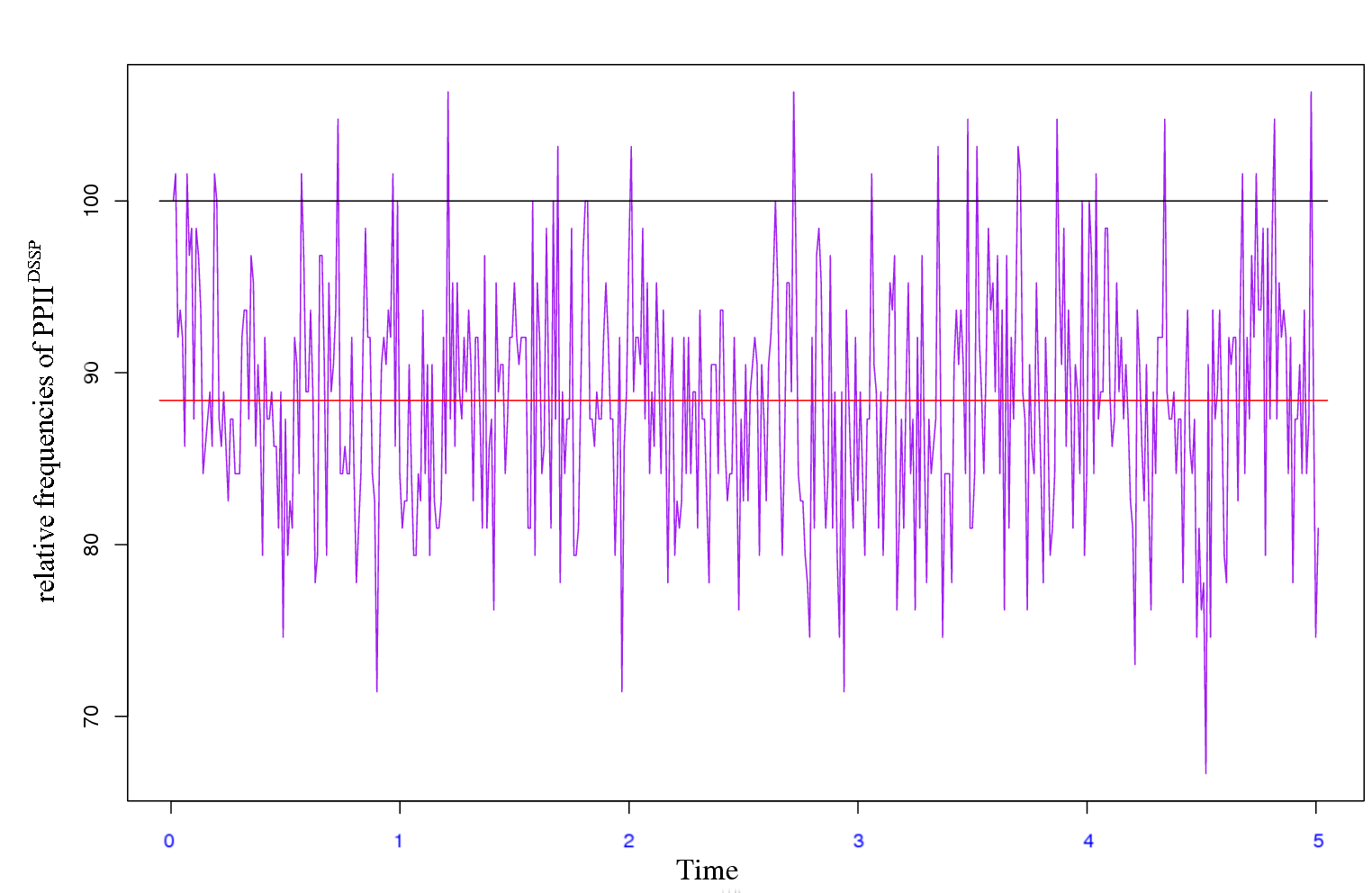


**Figure S7.2.** Relative frequency of PPII^DSSP^ assignment during the MD, the black line represents the frequency of the minimised structure and the red one, the average frequency.


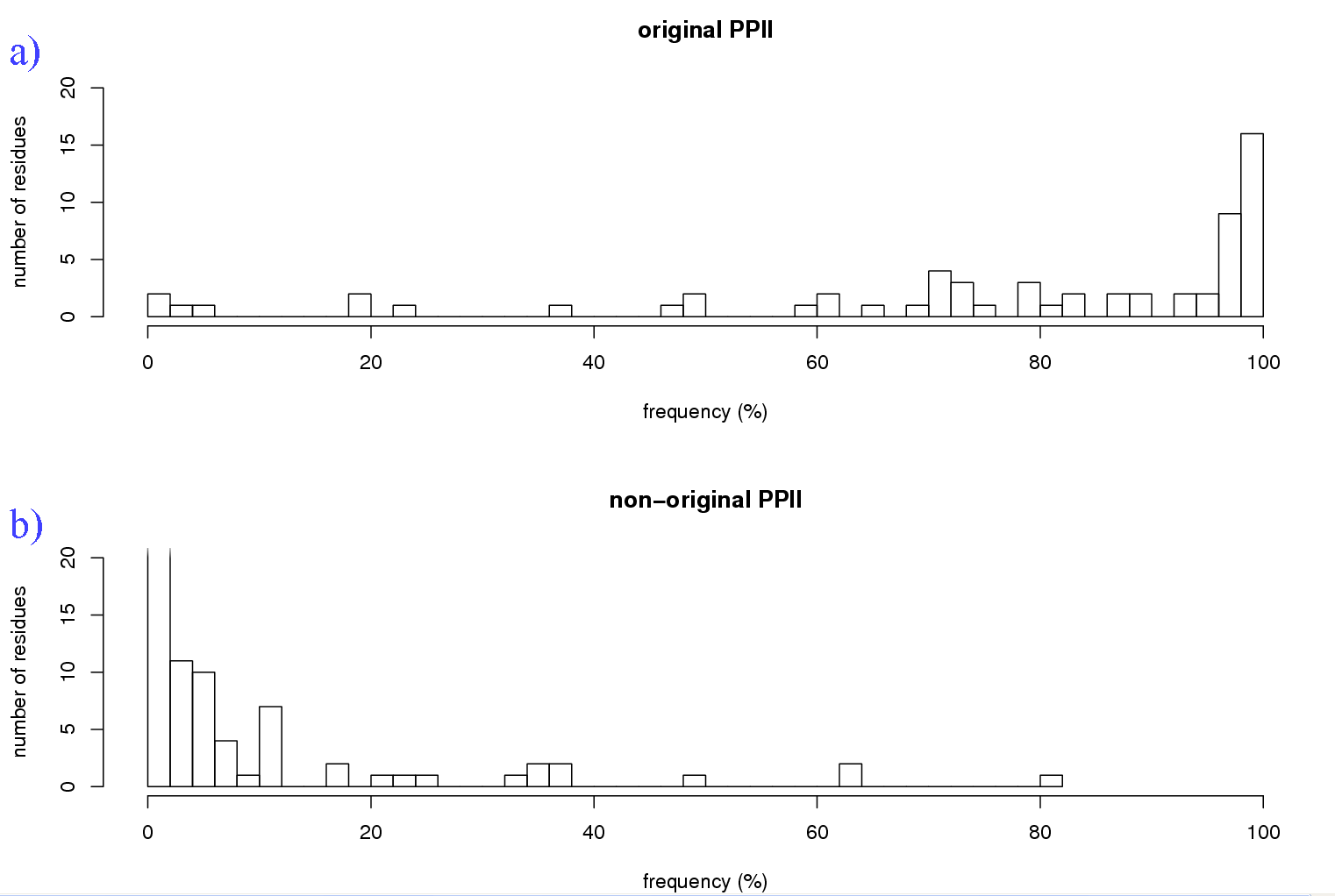


**Figure S7.3.** (a) Residues assigned as PPII^DSSP^ and (b) as non- PPII^DSSP^ percentage of times assigned as PPII^DSSP^.

Interestingly, a majority of PPII^DSSP^ residues stays associated to PPII^DSSP^ state during the simulation. Only 17% of residues show a frequency of PPII^DSSP^ less than 50% (see Figure S7.3a) and they shift to coil state mainly. Interestingly, some residues initially not associated to PPII^DSSP^ transit to PPII^DSSP^ state during the simulation. For few of them, it becomes the preferred state (3 residues are more than 50% associated to PPII^DSSP^), and 2.5% in more than 20% associated to PPII^DSSP^ (see Figure S7.3b). Figure 11a summarizes this information.


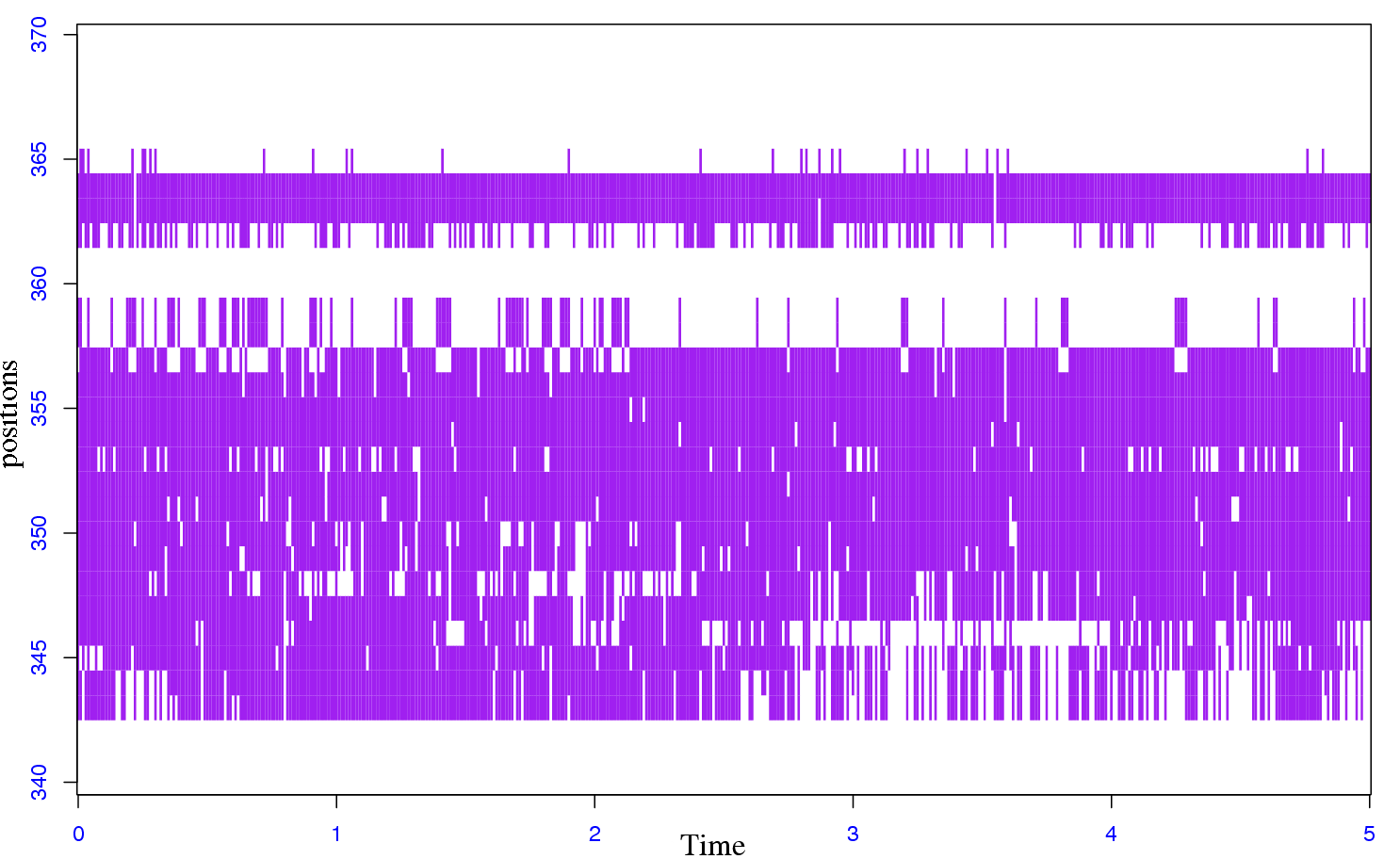


**Figure S7.4.** PPII^DSSP^ assignment of loop 340-370 during the MD, PPII^DSSP^ are in purple.

Figure 11b summarizes the PPII^DSSP^ frequency observed for region 340-370 (see Supplementary material 8b and 8c for a visualization of the dynamics). Figure S7.4 shows the same information as a function of time. As expected, the largest changes are located mainly at N and C caps of the loop. Nonetheless, it must be noted that Ncap region shrinks quickly from 345 to 343 and stays surprisingly well conserved along the simulation.

**References**

Berendsen, H.J.C., Postma, J.P.M., van Gunsteren, W.F., Di Nola, A., and Haak, J.R. 1984. Molecular dynamics with coupling to an external bath. *J. Chem. Phys.* **81:** 3684–3690.

Berendsen, H.J.C., van der Spoel, D., and van Drunen, R. 2005. GROMACS: A message-passing parallel molecular dynamics implementation. *Comp. Phys. Comm.* **91:** 43-56.

Berman, H.M., Westbrook, J., Feng, Z., Gilliland, G., Bhat, T.N., Weissig, H., Shindyalov, I.N., and Bourne, P.E. 2000. The Protein Data Bank. *Nucleic Acids Res* **28:** 235-242.

Cubellis, M.V., Cailliez, F., and Lovell, S.C. 2005. Secondary structure assignment that accurately reflects physical and evolutionary characteristics. *BMC Bioinformatics* **6 Suppl 4:** S8.

Hess, B., Bekker, H., Berendsen, H.J.C., and Fraaije, J.G.E.M. 1997. LINCS: a linear constraint solver for molecular simulations. *J. Comput. Chem.* **18:** 1463–1472.

Hess, B., Kutzner, C., van der Spoel, D., and Lindahl, E. 2008. GROMACS 4: Algorithms for highly efficient, load-balanced, and scalable molecular simulation. *J. Chem. Theor. Comp.* **4:** 435-447.

Jorgensen, W.L., Chandrasekhar, J., Madura, J.D., Impey, R.W., and Klein, M.L. 1983. Comparison of simple potential functions for simulating liquid water. *J. Chem. Phys.* **79:** 926-935.

Jorgensen, W.L., Maxwell, D.S., and Tirado-Rives, J. 1996. Development and Testing of the OPLS All-Atom Force Field on Conformational Energetics and Properties of Organic Liquids. *J. Am. Chem. Soc.* **118:** 11225–11236.

King, S.M., and Johnson, W.C. 1999. Assigning secondary structure from protein coordinate data. *Proteins* **35:** 313-320.

Kutter, S., Weiss, M.S., Wille, G., Golbik, R., Spinka, M., and Konig, S. 2009. Covalently bound substrate at the regulatory site of yeast pyruvate decarboxylases triggers allosteric enzyme activation. *J Biol Chem* **284:** 12136-12144.

Lindahl, E., Hess, B., and van der Spoel, D. 2001. GROMACS 3.0: A package for molecular simulation and trajectory analysis. *J. Mol. Mod.* **7:** 306-317.

Srinivasan, R., and Rose, G.D. 1999. A physical basis for protein secondary structure. *Proc Natl Acad Sci U S A* **96:** 14258-14263.

Tironi, I.G., Sperb, R., Smith, P.E., and van Gunsteren, W.F. 1995. Generalized reaction field method for molecular dynamics simulations. *J. Chem. Phys.* **102:** 5451–5459.

van der Spoel, D., Lindahl, E., Hess, B., Groenhof, G., Mark, A.E., and Berendsen, H.J.C. 2005. GROMACS: Fast, Flexible and Free. *J. Comp. Chem.* **26:** 1701-1718.
