# Supplementary material for: Assignment of PolyProline II Conformation and Analysis of Sequence – Structure Relationship
Source: PLoS One. 2011 Mar 31;6(3):e18401. doi: 10.1371/journal.pone.0018401 (PMC3069088; doi:10.1371/journal.pone.0018401)
Supplement: Figure S8 — Molecular dynamics of Saccharomyces cerevisiae pyruvate decarboxylase (PDB code 2VK8). [animation] (DOC) [file pone.0018401.s008.doc]

**Figure S8.** *Molecular dynamics of Saccharomyces cerevisiae pyruvate decarboxylase (PDB code 2VK8)*.

Visualization of the simulation can be found at <http://www.dsimb.inserm.fr/~debrevern/PII>.
